# First terror bird footprints reveal functionally didactyl posture

**DOI:** 10.1038/s41598-023-43771-x

**Published:** 2023-09-30

**Authors:** Ricardo N. Melchor, Silverio F. Feola, M. Cristina Cardonatto, Nahuel Espinoza, Manuel A. Rojas-Manriquez, Lorena Herazo

**Affiliations:** 1 Instituto de Ciencias de la Tierra y Ambientales de La Pampa, Universidad Nacional de La Pampa and Consejo Nacional de Investigaciones Científicas y Técnicas, Santa Rosa, La Pampa Argentina; 2 Departamento de Geología, Facultad de Ciencias Exactas y Naturales, Universidad Nacional de La Pampa, Santa Rosa, La Pampa Argentina; 3 Departamento de Geología, Universidad Nacional del Sur, Av. Alem 1253, 8000 Bahía Blanca, Argentina; 4 LA. TE. Andes S.A., Las Moreras 510, Vaqueros, Salta, Argentina

**Keywords:** Evolution, Palaeontology, Sedimentology

## Abstract

Terror birds (Aves, Phorusrhacidae) comprise the most outstanding group of South American Cenozoic avifauna, and have been considered dominant predators. Terrestrial habits were inferred using the reduction of their forelimbs and high body mass. Phorusrhacids were considered functionally tridactyl with three relatively short digits II–IV and a small, elevated digit I. The function of the ungual phalanges of digit II have been debated, including the utility of the ungual for retention or stabbing of prey. Incomplete or lack of preservation of foot bones have hampered understanding of the evolution and diversification of Phorusrhacidae. Here we show the first known and well-preserved footprints of Phorusrhacidae with a didactyl posture, which are named *Rionegrina pozosaladensis* igen. et isp. nov. These footprints yield unprecedented information on the locomotor habits of the group. The finding implies that medium-sized, Late Miocene (~ 8 Ma) phorusrhacids developed strong cursorial adaptations; achieved through reduction of digit II, raised metatarso-phalangeal pad, main body support in a large and thick digit III, and digit IV as outrigger. Raised and long claw of digit II was probably used in pining of prey. Phorusrhacid footprints differ from the Early Cretaceous didactyl footprints of deinonychosaurian dinosaur affinity by its larger size and strong mesaxony.

## Introduction

Terror birds (Aves, Phorusrhacidae) comprise the most outstanding group of South American Cenozoic avifauna, and have been considered as dominant predators in Cenozoic ecosystems^1–7^. Their terrestrial habits have been well established based on the reduction of their forelimbs and high body mass^1–3,8,9^. One of the pillars for the hypothesis of a predatory mode of life for phorusrhacids is based on the morphology of their hind limbs, which seem to be suitable for pursuing prey^10^. Comparison of the hindlimb of phorusrhacids with those of extant groups suggest that Mesembriornithinae and Patagornithinae were likely cursorial birds, but members of the Psilopterinae were likely walkers and waders^10^. Phorusrhacids have three relatively short digits II–IV^11,12^ and a small, elevated digit I, which is a feature related with terrestrial habits, and are thus considered tridactyl after Raikow^13^. The function of the ungual phalanges of phorusrhacids have been debated, including the utility of the ungual of digit II for retention of prey^11^, in a similar way to extant seriemas^14^, whereas other authors argued that large, curved and laterally compressed claws, is consistent with the function of stabbing prey^4^. The strength of the bones of some selected species can also imply kicking behavior to incapacitate their prey^4^. In spite of recent advances, the evolution, diversification and extinction of Phorusrhacidae are still debated issues^3,10,15,16^.

The purpose of this work is to infer locomotor habits of medium-sized phorusrhacids from the Neogene of Patagonia, using excellently preserved footprints recently found in coastal outcrops of the Río Negro Formation. These are the first and only known fossil footprints assigned to this group of Aves.

Sedimentary rocks hosting the studied footprints belong to the Upper Miocene-Lower Pliocene Río Negro Formation at the San Matías Gulf (Atlantic coast of Argentina), where the best exposures are in several tens of kilometers of coastal cliffs west of the Negro River mouth^17^. The Río Negro Formation is typified by greyish-blue cross-bedded sandstone packages alternating with finer grained lenses deposited in aeolian dune, wet and dry interdune, fluvial, tidal flat, shoreface and offshore marine settings^17–21^. Three members have been distinguished in the Río Negro Formation from the exposures of the Atlantic coast of the Río Negro Province^17^. The lower and upper members are continental and the middle member is of marine origin. Available absolute ages for the Río Negro Formation are 6.78 Ma^22^ (Sr isotopic relationships from bivalves) for the top of the middle member, and 4.41 ± 0.5 Ma^23^ for a tuff bed of the upper member (fission track dating method). The middle member is considered as part of the Paranense transgression^22^.

## Results

### Paleoenvironmental setting and age

**Figure 1:**
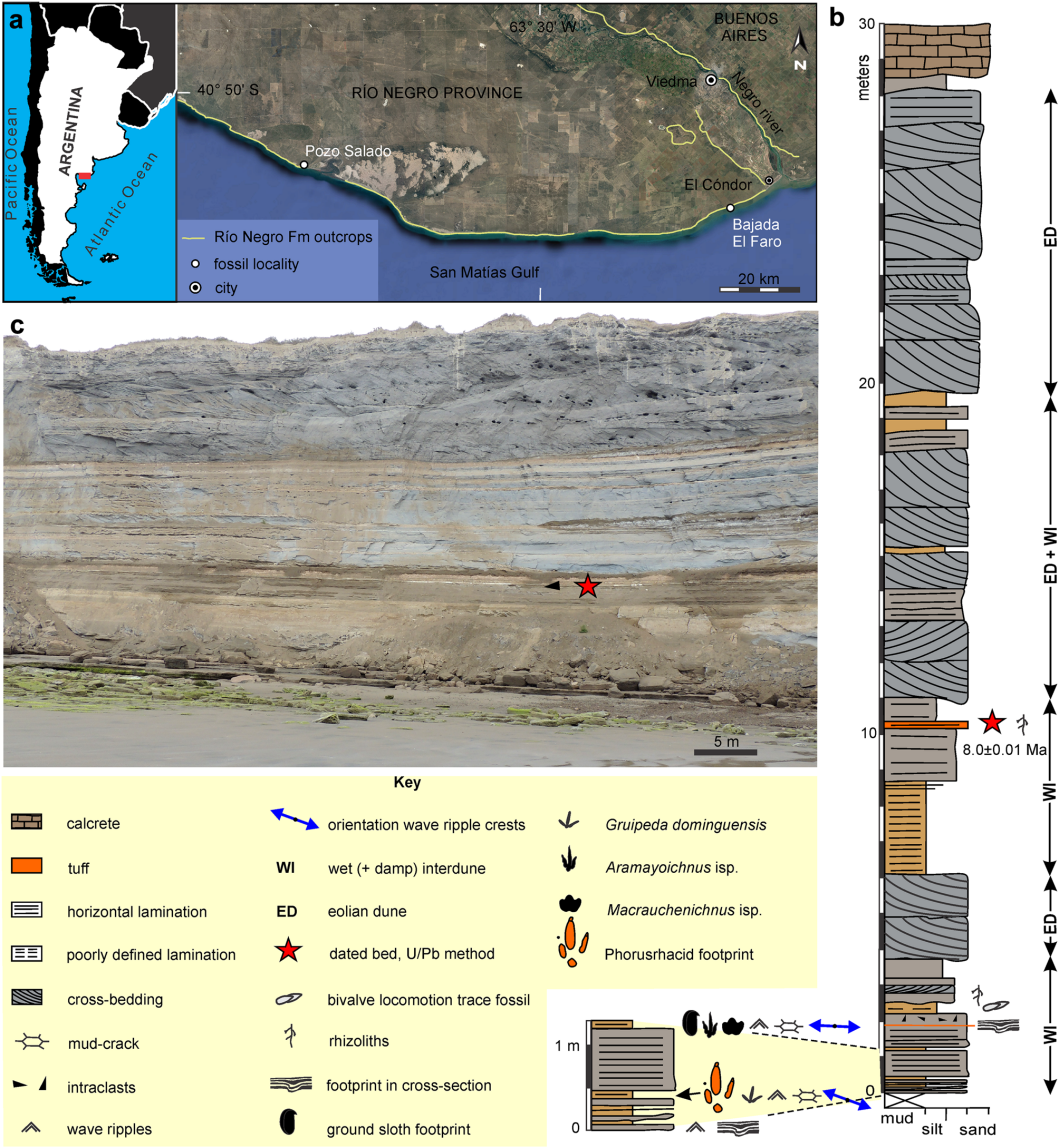
Location map and stratigraphic section of the Río Negro Formation at Pozo Salado locality (Río Negro Province, Argentina). (**a**) Location of the study area in Argentina (inset) and distribution of outcrops of the Río Negro Formation. Image generated in Google Earth Pro and then exported to Corel Draw X8. (**b**) Stratigraphic section of the Río Negro Formation at Pozo Salado locality (41° 00′ 49.45″ S; 64° 09′ 54.17″ W) with dated tuff level and position of phorusrhacid footprints. (**c**) View of sea-cliff exposure of the Río Negro Formation at the study locality. The arrow indicates the dated tuff level.

The fossil footprints were identified at the Pozo Salado locality (41° 00′ 49.45″ S; 64° 09′ 54.17″ W) (Fig. 1a). The local succession of the Río Negro Formation is at least 30 m thick (Fig. 1c) with no exposed base and includes eolian and associated lacustrine deposits (Fig. 1b). Phorusrhacid footprints occur in the lowermost part of the local sequence (WI, Fig. 1c), in particular, on top of an interval composed of thin-bedded sandstone interbedded with thin reddish mudstone showing wave ripples and occasional mud-cracks. Wave ripple crests with an average orientation of N78°W occur associated with small shorebird-like footprints (*Gruipeda dominguensis*). The two overlying beds are thicker and composed of parallel-laminated well-sorted sandstone and interbedded mudstone beds. The top of these beds contains fossil footprints attributed to ground sloths, Rheidae (*Aramayoichnus* isp.) and Macraucheniidae (*Macrauchenichnus* isp.). The uppermost mudstone interval contains bivalve locomotion trace fossils and is capped by a sandstone bed with rhizocretions. This lowermost interval containing the phorusrhacid footprints is interpreted as a shallow lacustrine setting with development of wet to moist mudflats that were the adequate medium for registration and preservation of tetrapod footprints. The lake mudflats were flooded and exposed repeatedly, with the subsequent deposition of mud and production of subaqueous bivalve locomotion trace fossils; and alternating periods of exposure, desiccation and eventual rooting by plants. Local orientation of the lake coast was roughly N78°W, as inferred from dominant wave ripple crest orientation.

The overlying section represents an alternance of thick cross-bedded, well-sorted sandstone packages and reddish mudstone intervals. They are interpreted as eolian dunes (Fig. 1b) and interdune lakes (WI in Fig. 1b) that composed the main paleoenvironmental setting. Eolian dunes dominate toward the top of the section. A 0.15 m thick parallel- and cross-laminated light-gray vitric tuff bed from the top of the second lacustrine interval yielded an age of 8.0 ± 0.1 Ma (U/Pb method on zircon) (Fig. S1 and Table S1). In consequence, the phorusrhacid footprints are dated as Tortorian (Late Miocene).

### Systematic ichnology

Ichnogenus *Rionegrina* igen. nov.

Type ichnospecies *Rionegrina pozosaladensis* isp. nov.

#### Etymology

After the Río Negro Province and the homonymous geologic formation, where this ichnogenus occurs.

#### Diagnosis

Functionally didactyl footprints of moderate to large size (footprint length > 300 mm) composing a bipedal trackway. Footprints markedly mesaxonic with footprint length /width ratio ~ 1.5. Thick, straight, deep and fusiform digit III impression, smaller slightly curved digit IV, and digit II composing a short and shallow elliptical impression. Digit impressions commonly lacking digital pads and not united to a shallow subcircular metatarso-phalangeal pad impression. Well-developed and thick subtriangular claw trace in digit impressions III and IV. Tip of claw imprint II disconnected from digit impression occasionally preserved. Average divarication II–III smaller than III–IV. Narrow trackway (external trackway breadth/footprint width ~ 1.6) with high pace angulation (> 160°), and negative (inward) rotation of footprints. Differs from *Velociraptorichnus* by a much larger size, marked mesaxony, and separation between digital and metatarso-phalangeal pads. Distinguished from *Dromaeopodus* by larger III–IV divarication, longer and straight digit III impression, lack of well-defined phalangeal pads, and proportionally smaller metatarso-phalangeal impression. Differences with *Dromaeosauripus* are a much larger size, presence of a metatarso-phalangeal pad impression, straight digit III impression that is much larger than that of digit IV, and absence of well-marked phalangeal pad imprints.

#### Remarks

**Figure 2:**
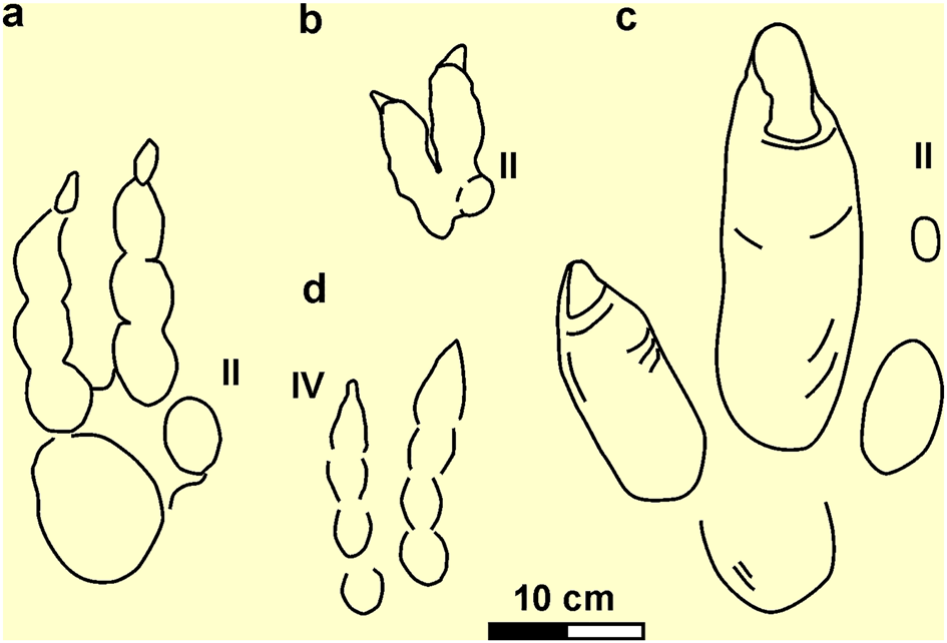
Comparison of former didactyl ichnogenera with *Rionegrina* igen. nov. (**a**) *Dromaeopodus*^25^. (**b**) *Velociraptorichnus*^24^. **c**, *Rionegrina* igen. nov. (**d**) *Dromaeosauripus*^26^. II = digit II, IV = digit IV.

*Rionegrina* exhibits a set of morphological features that differs from any named ichnotaxa and previously recorded fossil or extant footprint. Comparisons with morphologically similar didactyl ichnogenera are related to Early Cretaceous footprints of deinonychosaurian (theropod dinosaur) affinity. These includes *Velociraptorichnus*, *Dromaeopodus,* and *Dromaeosauripus*^24–26^ (Fig. 2a,b,d)*. Velociraptorichnus* and *Dromaeopodus* share a “heel” impression and a reduced impression of digit II, whereas *Dromaeosauripus* is composed of nearly parallel impressions of digits III and IV, with no “heel” impression^27^ (Fig. 2a,b,d). *Rionegrina* is about 30% larger than the largest specimens of *Dromaeopodus* (the largest ichnotaxon of deinonychosaurian affinity), display a marked mesaxony whereas the potentially comparable ichnogenera have digit III and IV of similar length (Fig. 2c). In addition, *Rionegrina* have large divarication III-IV angle, lack of well-defined phalangeal pads (or a single well-defined digital pad) and have straight digit impressions. Other ichnogenera of purported didactyl theropods are excluded from this comparison: *Menglongipus, Paravipus,* and *Sarmientichnus*^28–30^*. Menglongipus* is based on material of suboptimal preservation^27^ and thus of dubious validity. *Paravipus* is a Jurassic purported didactyl ichnogenus that probably represent swim trace fossils^27^. S*armientichnus* is also a Jurassic ichnogenus representing didactyl theropod footprints reflecting particular substrate conditions^31^ that was recently considered of deinonychosaurian affinity^32^.

**Figure 3:**
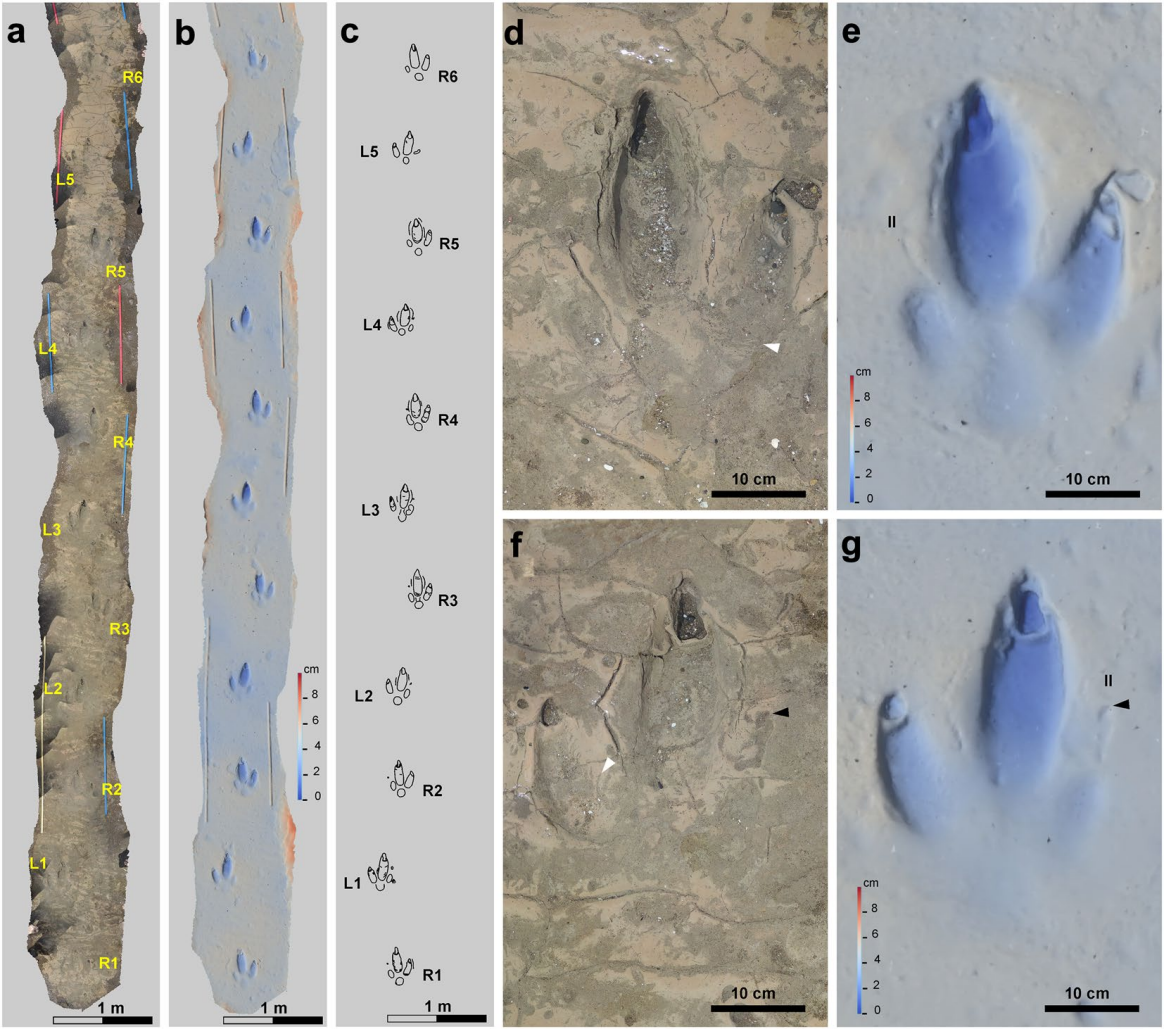
Holotype trackway of *Rionegrina pozosaladensis* igen. et isp. nov. (**a**–**c**) Orthomosaic, digital elevation model and interpretative drawing of the holotype. (**d**,**e**) Detail of orthomosaic and digital elevation model of fifth right footprint (R5). (**f**,**g**) Detail of orthomosaic and digital elevation model of the fourth left footprint (L4). White arrow points to wrinkle traces and black arrows to tip of digit II claw trace. R1-R6: consecutive right footprints, L1–L5: consecutive left footprints, II: digit II.

**Figure 4:**
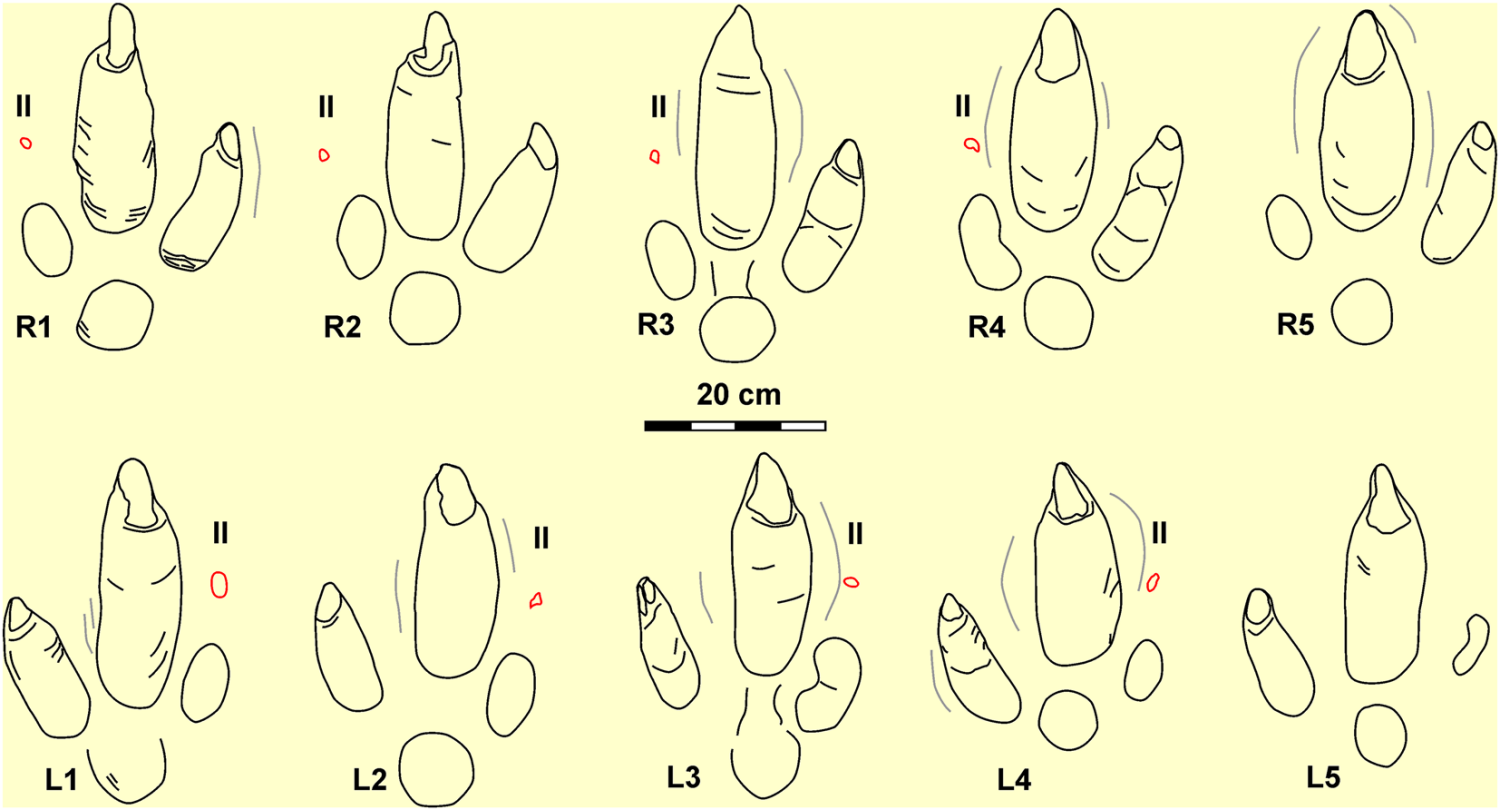
Detailed line drawing of individual right (above) and left (below) footprints of the holotype trackway of *R. pozosaladensis*. Grey lines are marginal ridges and red lines represent the tip of claw trace of digit II. R1–R5: consecutive right footprints, L1–L5: consecutive left footprints, II: digit II.

*Rionegrina pozosaladensis* isp. nov. (Figs. 3, 4).

#### Etymology

After the Pozo Salado locality, Río Negro Province, Argentina; where this ichnospecies was found.

#### Holotype

In situ trackway composed of eleven consecutive footprints (R1 to R6, Fig. 3a–c). Orthomosaic (http://dx.doi.org/10.6084/m9.figshare.23749107), digital elevation model (http://dx.doi.org/10.6084/m9.figshare.23749260) and interpretative drawing (http://dx.doi.org/10.6084/m9.figshare.23751186) of complete trackway. A 3D printing of a left (L1) and right (R2) footprint of the holotype trackway is housed as specimens GHUNLPam 29796 and 29797, at the Colección Paleontológica de la Facultad de Ciencias Exactas y Naturales, Universidad Nacional de La Pampa (Santa Rosa, La Pampa, Argentina).

#### Diagnosis

Only known ichnospecies, same as for the ichnogenus.

#### Description

**Figure 5:**
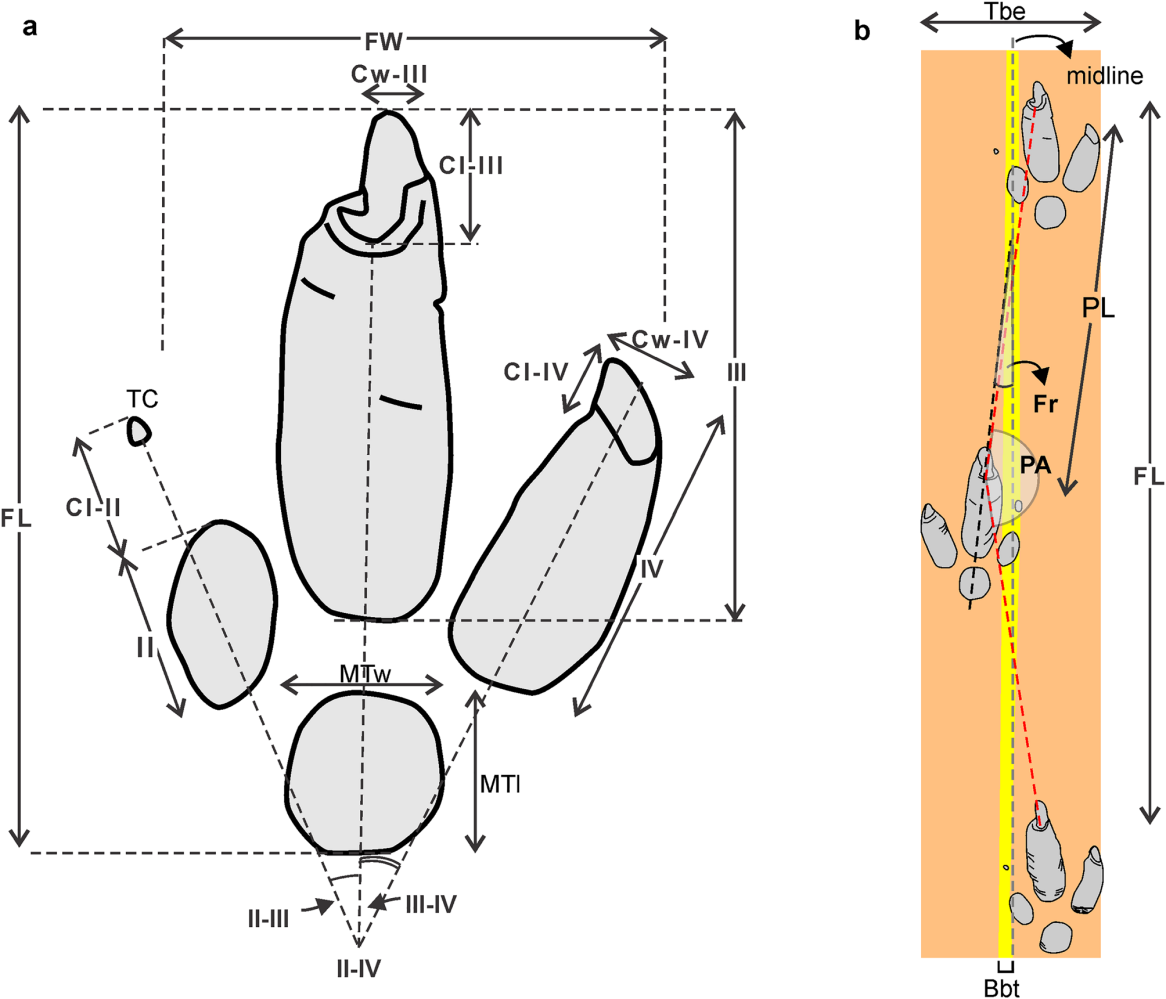
Measurements on fossil footprints. *FL* footprint length, *FW* footprint width; II, III, IV: length of digits II to IV; MTl, MTw: length and width of metatarso-phalangeal pad; II-IV: total divarication; II–III, III–IV: angle between digits II and III and between III and IV, respectively. Cl-II: length of claw II; TC: tip of claw II; Cl-III, Cw-III: length and width of claw of digit III; Cl-IV, Cw-IV: length and width of claw of digit IV; *PA* pace angulation; *PL* pace length, *Fr* footprint rotation with respect to the midline, *Tbe* external trackway breadth, *Bbt* breadth between tracks.

Nearly straight trackway composed of seventeen consecutive footprints preserved as negative epirelief with overall orientation of N187°. Eleven consecutive footprints (R1 to R6) display a very good preservation (grade 3 or 2 of the preservational scale^33^) and intersect wave ripples (Fig. 3a–c). Of the remaining footprints, L6 and L7 are missing and R9 is incomplete. Mud-cracks project from the surrounding surface into the footprints and, in some cases, follow the boundary of digit III impression (Fig. 3d). Mud-cracks were not recorded intersecting footprints. Footprints registered in a thin reddish mudstone lamina with wave ripples and mud-cracks (the later are wider toward the end of the trackway). Measurements on the studied material were taken according to the conventions described in Fig. 5 and summarized in Table S2 (Supplementary Material). Large (average length = 371.45 mm, width = 253 mm) functionally didactyl footprints with moderate elongation (average FL/FW = 1.47), marked impressions of digit III (up to 57 mm deep), then of digit IV, and shallower metatarso-phalangeal pad and digit II impressions (Figs. 3f,g, 4). Digit impressions are not connected to the metatarso-phalangeal impression, except for some footprints that exhibit a shallow “bridge” with digit III. Impression of digit III is fusiform, the longest (average = 250 mm) and thickest (average width = 88 mm) with marginal ridges on the medial and lateral sides, and large (average length = 71.45 mm, width 33.09 mm) and deeply set subtriangular claw traces (Fig. 3d–g). Digit IV impression is the second longest (average length = 163.42 mm, average width = 60.33 mm). with a slightly curved external margin and a smaller claw trace (average length = 30.54 mm, width = 27.90 mm). The elliptical digit II impression is the shortest (average length = 85.09 mm, width = 45.46 mm), shallower and exhibits an elliptical to subtriangular imprint (about 12 mm in diameter) located ~ 72 mm in front of the digit impression that is a probable claw imprint (Fig. 3f–g). Subcircular to pear-shaped metatarso-phalangeal pad impression (73.3 mm by 75.6 mm) is shallow. Transverse to oblique wrinkles occur inside digit impressions III, IV and the metatarso-phalangeal pad impression (Fig. 3d,f). The trackway is straight and have an average pace angulation of 168.8°, pace length of 918 mm, and stride length of 1830 mm. Individual footprints display a negative (inward) rotation averaging 6.8°, the external trackway width is 407.8 mm and the breadth between tracks is negative (average 75.8 mm) (Fig. 3a–c).

#### Remarks

**Figure 6:**
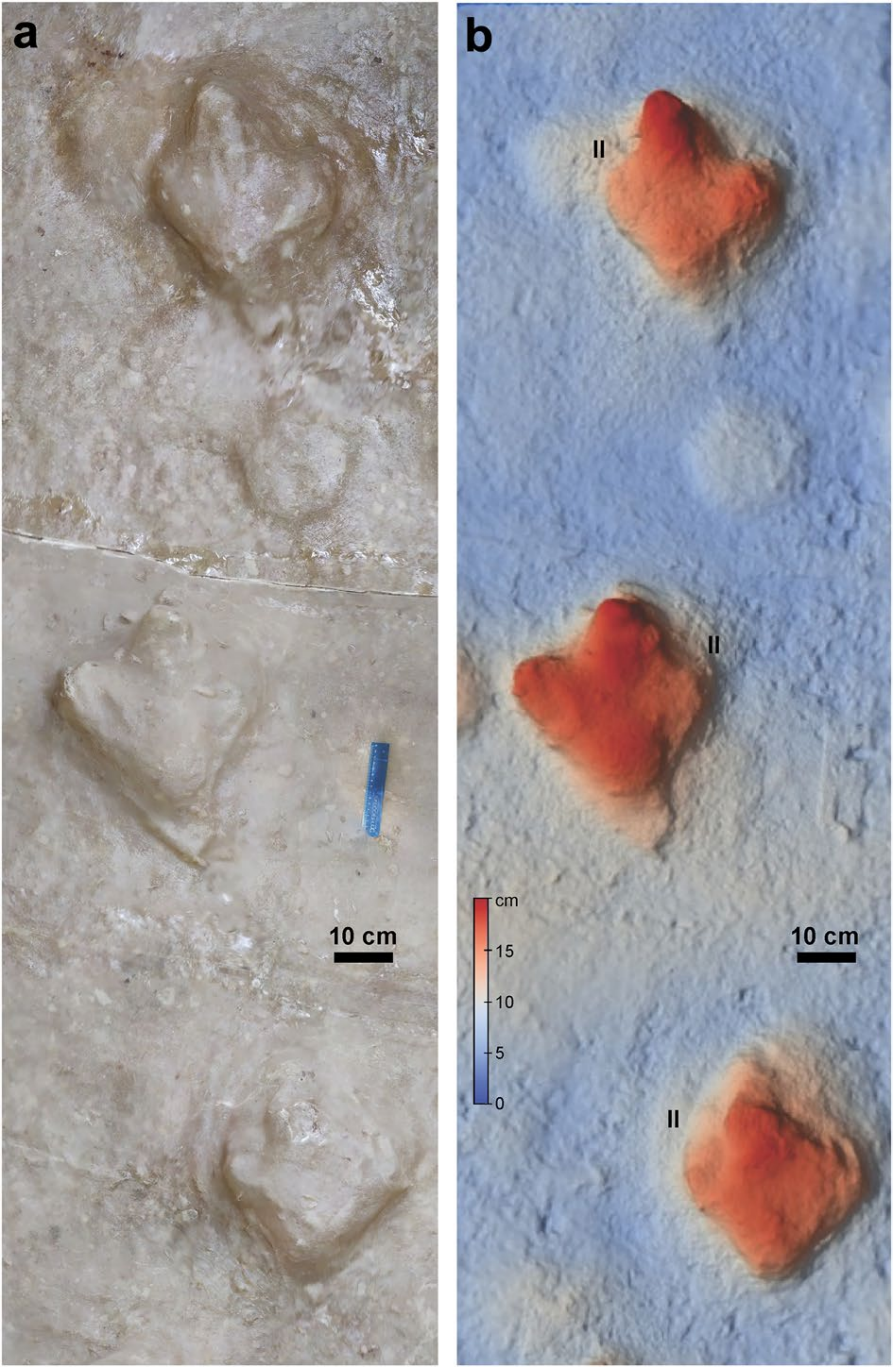
Footprints from the Río Negro Formation formerly compared with Cariamidae in Ref.^34^. Three consecutive deep undertracks preserved as positive hyporelief in a fallen block of sandstone. Specimen P.ICH.UNS 102 A (polyester resin replica) from the Departamento de Geología, Universidad Nacional del Sur, Bahía Blanca, Argentina. (**a**) Orthomosaic. (**b**) Digital elevation model. II: digit II impression.

Three consecutive tridactyl footprints with a moderate reduction of digit II and composing a bipedal trackway has been reported for the unit, and compared with the footprints of large birds (cf. Cariamidae)^34^. These are deep undertracks (about 0.15 m deep) of roughly comparable size (340 mm long, 305 mm wide) that are proportionally wider and lack the claw trace of digit III (Fig. 6a, b). This material is considered of dubious affinity and distinct from *R*. *pozosaladensis*.

### Geometric morphometric analysis

**Figure 7:**
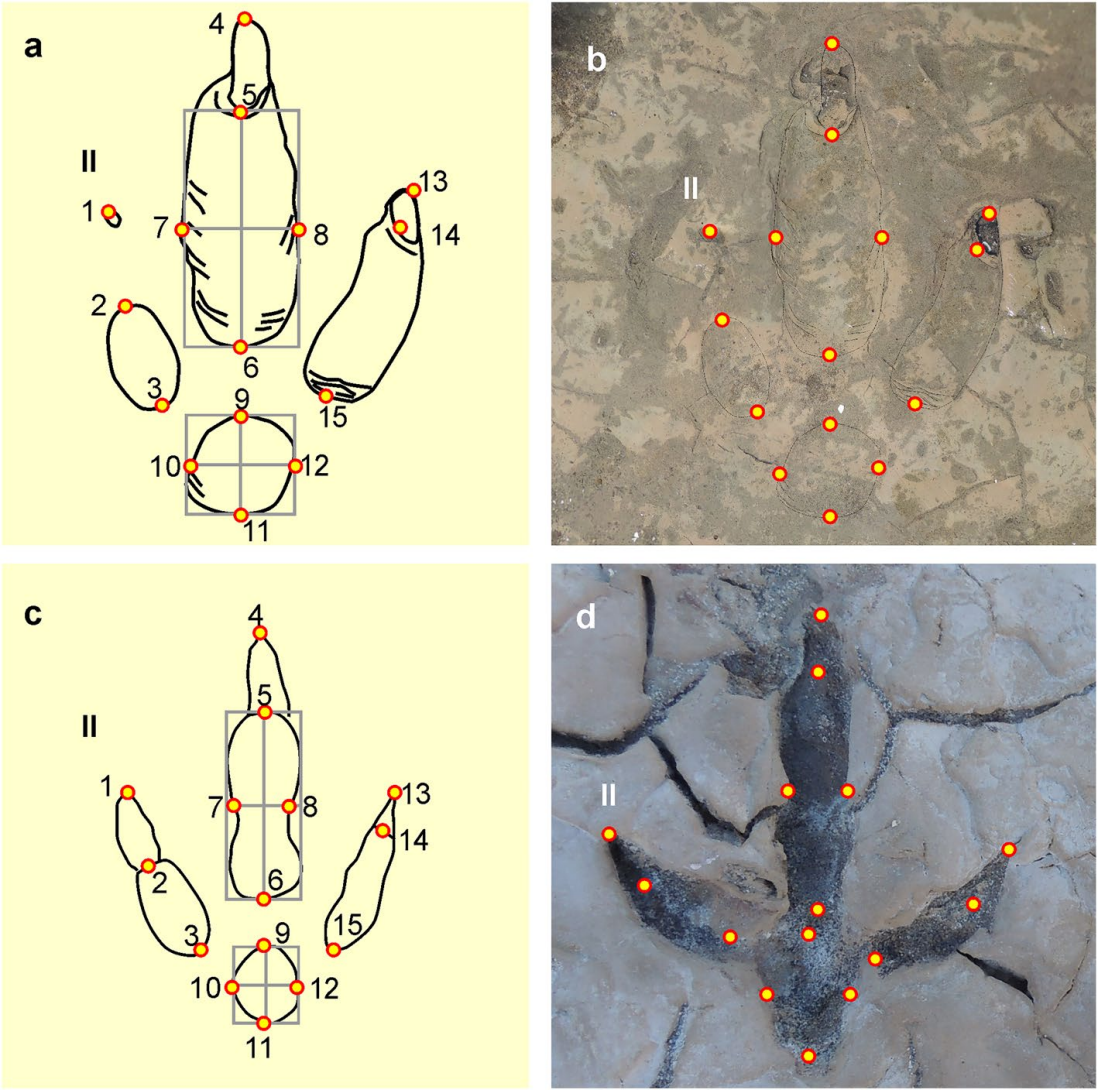
Landmarks used in geometric morphometric analyses. (**a**,**b**) Placement of landmarks on *Rionegrina pozosaladensis* igen. et isp. nov. (**c**,d) Landmarks used in extant and fossil Rheidae footprints.

**Figure 8:**
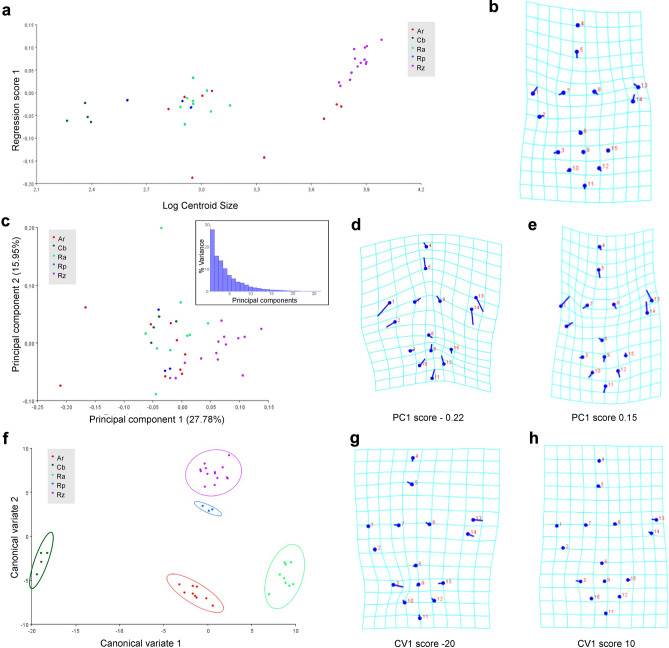
Geometric morphometric plots comparing *Rionegrina pozosaladensis* igen. et isp. nov. (Rz), with *Aramayoichnus rheae* (Ar, fossil rheid footprints), and footprints of extant *Rhea americana* (Ra), *Rhea pennata* (Rp), and *Chunga burmeisteri* (Ch) (n = 39). The numbered dots indicate the landmark locations in the mean shape of the sample; the sticks indicate the changes in the relative positions of the landmarks. (**a**) Multivariate regression of footprint shape onto size (as logarithm of centroid size) classified by (ichno)species. (**b**) Deformation grid reflecting shape changes. (**c**) Principal components plot of PC1 vs. PC2 of analysed footprints. The inset display the histogram of principal components. (**d**,**e**) Shape changes visualised by transformation grids at PC1 scores − 0.20 and 0.15. (**f**) Scatter plot showing the variation in shape of footprints of different ichno(species) along the first two canonical variate (CV1 and CV2) axes. The different ichno(species) represented by contrasting footprint shapes are clearly separated across CV1 (delimited by confidence ellipses). **g-h**, Shape changes, illustrated by the deformation grids, at CV1 scores -20 and 10, respectively.

Geometric morphometric comparison of *R*. *pozosaladensis*, *Aramayoichnus rheae* (fossil rheid footprints), and footprints of extant *Rhea americana*, *Rhea pennata* and *Chunga burmeisteri* were conducted using 15 landmarks (Fig. 7a–d). Supplementary Material Table S3 contains detailed information on the footprints used for comparison. Complete results of geometric morphometric analyses are included in Table S4 (Supplementary Material). Only 10.40% of the shape variation can be explained by size (p < 0.0002) (Fig. 8a, b). In the PCA, the first four components accounted for 67.74% of the total variation (Fig. 8c). The transformation grid at score -0.22 of the PC1 axis (Fig. 8d) shows the maximum variation related to digits II and IV (landmarks 1, 2, 13, 14), and the metatarso-phalangeal pad (landmarks 9–12) also contributes to a lesser extent. At PC1 score 0.15, the maximum variation is observed in a lower total digit divarication (landmarks 1, 2, 13 and 14), and size of digit III (landmarks 5, 7, 8) (Fig. 8e). The first two canonical variates accounted for 92.36% of the total variation of the tracks (Fig. 8f). *Rionegrina pozosaladensis* and the groups of extant and fossil rheid footprints used for comparison are clearly separated along the CV1 axis. Shape changes along CV1 axis were found related with digits III and IV and proximal part of digit II (landmarks 5–8, 13–15 and 3) at score −20, and tip of digit IV and proximal part of digit II at score 10 (landmarks 3, 13, and 14) (Fig. 8g,h). Mahalanobis and Procrustes distances obtained by pairwise comparisons of *R*. *pozosaladensis* with the remaining groups of fossil and extant footprints were significantly different (p ≤ 0.0007 and p ≤ 0.0027, respectively).

## Discussion

### Producer of *Rionegrina pozosaladensis*

Footprints are considered true tracks because contains wrinkle traces (related to integumentary imprints of the producer), display cross-cutting relationships with wave ripples, and mud-cracks follow their boundary. In consequence, it is possible to infer that footprints were registered in a lake mudflat after wave ripple formation and were later exposed and mud-cracked. The presence of wider mud-cracks toward the south, suggest that the producer was moving away from the lake coast. Considering a height at hip of 0.81 m^12^, the estimated speed of the footprint producer is 2.74 m/s, using the method by Alexander^35^.

**Figure 9:**
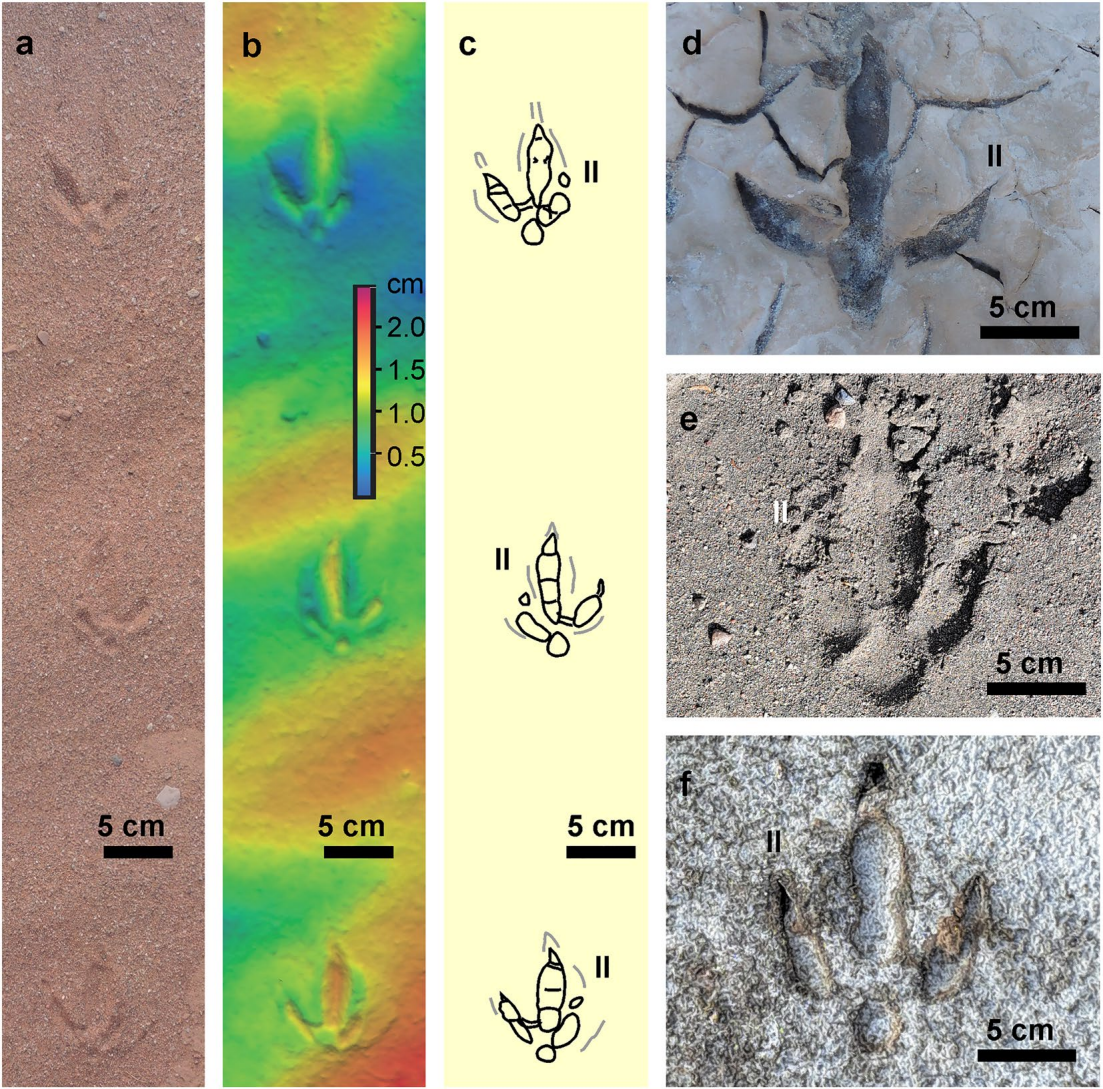
Examples of footprints of extant birds and fossil footprints used for morphological comparison with *R*. *pozosaladensis* (see also Supplementary Material Table S3). (**a–c**) Orthomosaic, digital elevation model and interpretative drawing of a portion of a trackway of *Chunga burmeisteri* in sand from the Talampaya National Park, La Rioja Province, Argentina. (**d**) *Aramayoichnus rheae* from Río Negro Fm. (Late Miocene), Bajada El Faro, Río Negro Province, Argentina. (**e**) Footprint of extant *Rhea pennata* in sand from La Amarga, Neuquén Province, Argentina. (**f**) Footprint of extant *Rhea americana* in mud from Salitral de La Perra, La Pampa Province, Argentina. II: digit imprint.

The overall morphology of the footprints, bipedal configuration of the trackway, and the age of the hosting rocks suggest that the potential producer should be a large bird that inhabited Patagonia in the Late Miocene. The average body mass of the avian producer is estimated at about 55 kg using an allometric equation related to the area of the footprints of birds and reptiles^36^ (Table S2). This body mass is likely to be underestimated as the database used for calculation^36^ lacks information on birds larger than 22 kg. Potential candidates from the Miocene fauna of South America are Cariamidae, Rheidae and Phorusrhacidae. The Cariamidae is represented since the Early Miocene by small bodied specimens, with a size similar to modern seriemas^37^. Modern *C. burmeisteri* footprints are roughly similar to *R*. *pozosaladensis* in the overall configuration, including reduction of digit II, although are considerably smaller (Fig. 9a–c). Miocene rheids have a tridactyl feet and are mostly smaller than the extant *Rhea americana* (that is about 23 kg^38^), especially the fossil species of *Rhea*^39^*,* although *Opisthodactylus* is slightly larger (Table S5). *Opisthodactylus kirchneri* is considered about 10% larger than *R. americana* and of similar size to *Opisthodactylus patagonicus*^40^. Fossil rheid footprints occur in the Río Negro Formation (Fig. 9d) and were also recorded in other Neogene and Quaternary units from Argentina^41,42^. Fossil rheid footprints are tridactyl mesaxonic with a maximum length of about 170 mm and were mostly assigned to the ichnospecies *Aramayoichnus rheae*^41,42^. Miocene rheid footprints are very similar in morphology and size to extant *Rhea* footprints (Fig. 9e,f). Geometric morphometric comparison of *R*. *pozosaladensis*, *A. rheae*, and footprints of extant *R*. *americana*, *R. pennata* and *C*. *burmeisteri* suggests that the producer of *R*. *pozosaladensis* is not morphologically related to Cariamidae or Rheidae (Fig. 9).

**Figure 10:**
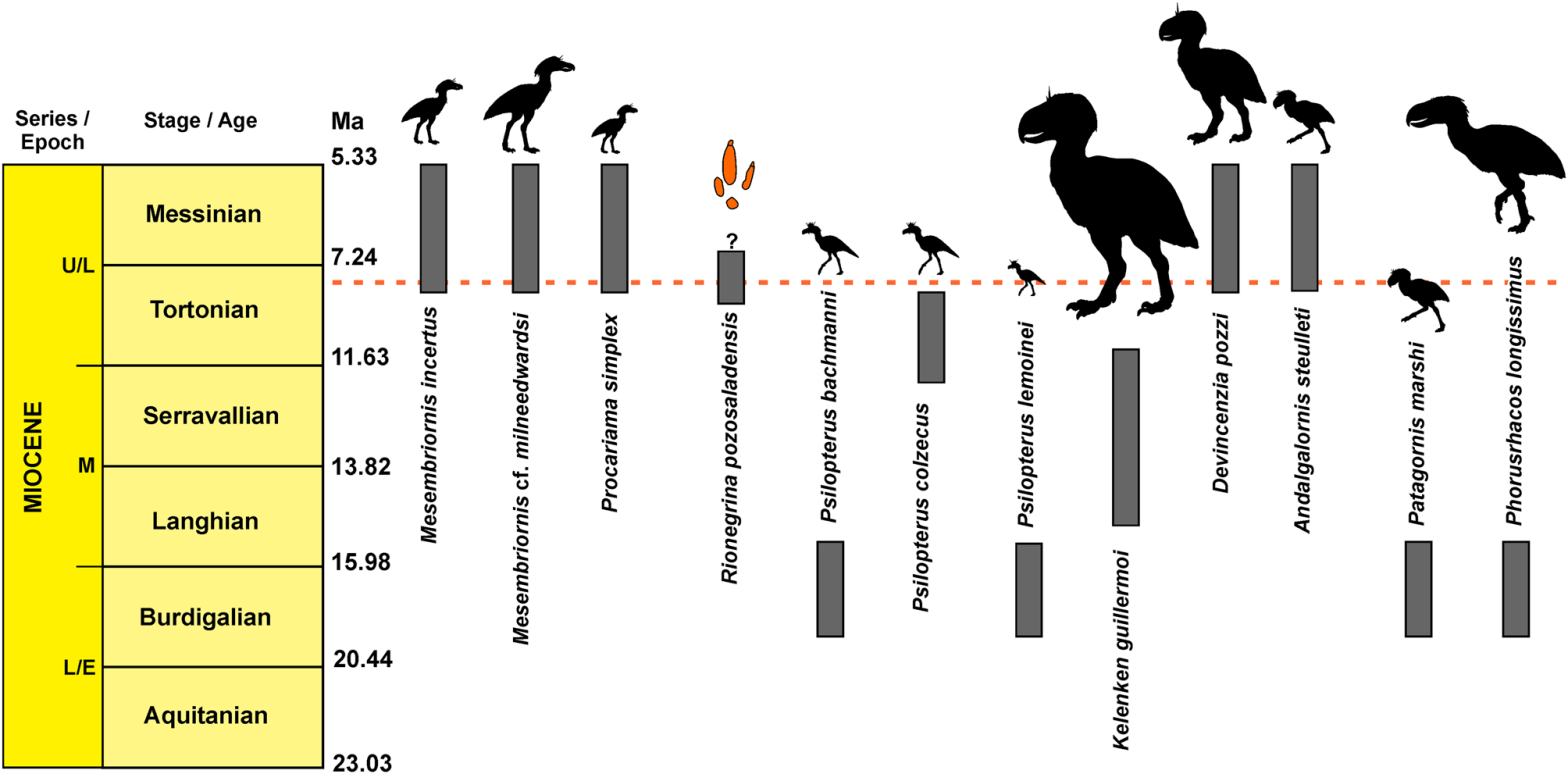
Stratigraphic distribution of Miocene Phorusrhacidae and *R*. *pozosaladensis* igen et isp. nov. Silhouettes after Ref.^15^ and time scale after https://stratigraphy.org/chart. Relative sizes of silhouettes using estimations of body mass summarised in Supplementary Material Table S6. The red dashed line represents the age of the dated tuff level from the study locality.

**Figure 11:**
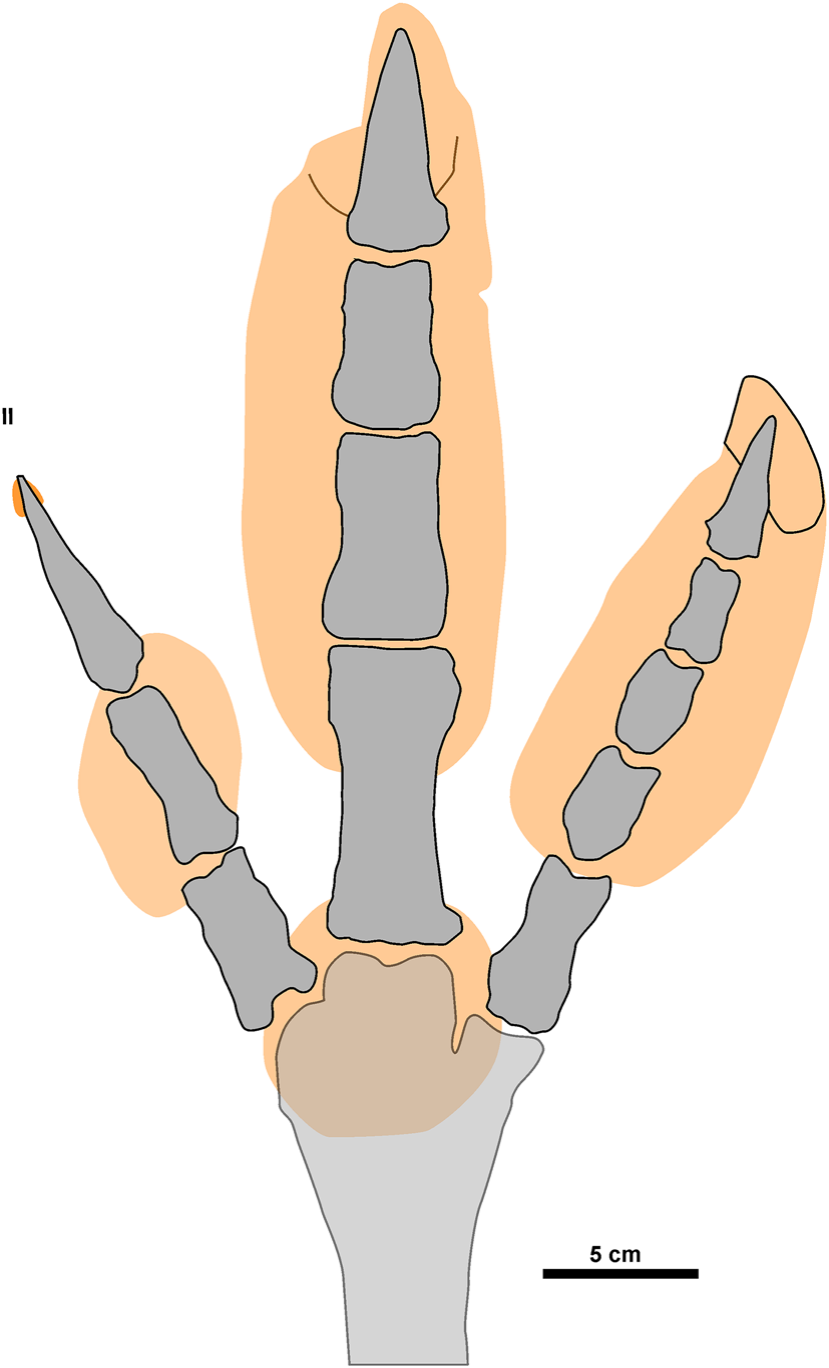
Tentative osteological reconstruction of the foot of the producer of *R. pozosaladensis* igen. et isp. nov. using the foot of *Mesembriornis incertus* after Ref.^10^.

South American Phorusrhacidae were recorded from the Middle Eocene to Early Pleistocene and included gracile to gigantic taxa^3,12,15,37,43^. For the Miocene, the taxa with intermediate body mass (~ 30 – 70 kg) are *Mesembriornis incertus*, *Mesembriornis* cf. *milneedwardsi*, *Andalgalornis steulleti* and *Patagornis marshi* (Table S6 and Fig. 10). In particular, the body mass estimates that are closest to the producer of *R. pozosaladensis* are those of *M*. *milneedwardsi*^12,44^ from the Lower Pliocene, although this taxon was also recorded in the Late Miocene^45^. On the basis of bone remains the weight of *M*. *milneedwardsi* was estimated at 53–66 kg^12,44^ (Table S6, Supplementary Material). Preservation of foot bones of phorusrhacids is commonly incomplete and most are represented by incomplete foots, isolated digits or loose phalanges. A nearly complete foot is only known for some phorusrhacids, like the medium to small bodied Mesembriornithinae (*M*. *incertus*)^10^ and Psiloptherinae (*Psilopterus* *colzecus*)^46^ and very large Physornithinae (*Paraphysornis brasiliensis*)^47^. Using for comparison digit III (estimated as the length of half of the first phalanx plus the length of the remaining phalanges) is considerably smaller than the digit III impression of *R*. *pozosaladensis* for *P*. *colzecus* (~ 40%), and *M. incertus* and *P. marshi* (~ 50%) (Fig. 11). Comparisons with the giant *Paraphysornis brasiliensis* (about 180 kg)^3^ is only possible considering digit IV, because digit III is incomplete. This comparison suggest that the length of digit IV (170 mm) is similar than the average length of the corresponding digit impression in *R*. *pozosaladensis* (163 mm). In consequence, it is likely that the body mass of the producer of *R*. *pozosaladensis* is larger than the estimated in this study. *Kelenken guillermoi* from the Middle Miocene of Patagonia is discarded as potential producer due to its gigantic size^5^, and is also older than *R*. *pozosaladensis* (Fig. 10).

To conclude, the most likely producer of *R*. *pozosaladensis* is a medium- to large-sized phorusrhacid, probably belonging to Mesembriornithinae, although no exact match with a known genus is possible to date. Cariamidae and Rheidae are discarded as candidates considering the Miocene fossil record, morphology of extant and fossil footprints, and body mass.

### Implications for locomotion of phorusrhacids

Phorusrhacidae autopodium has been commonly reconstructed as tridactyl mesaxonic with a longer digit III, and raised digit I. Ungual phalanges are sharp, arcuate and laterally compressed^2,3,11^, similar to those of Cariamidae^46^. Digit II exhibits a potential noticeable extension and a ungual that is more curved that those of the remaining digits, similar to Cariamidae^11^. Although phorusrhacids does not exhibit hyperextension of digit II as typical of deinonychosaurs^48^, it has never been shown the marked reduction of digit II and raised posture of the ungual phalange, as inferred from *R*. *pozosaladensis*. A cursorial habit has been inferred from the morphometry of pelvis and hindlimbs of some groups of Phorusrhacidae (Mesembriornithinae and Patagornithinae)^10^, and from biomechanical models of selected species^4^.

The marked reduction in digit II, large and deep impression of digit III with deep and large claw trace, and shallower digit IV in *R*. *pozosaladensis* can be interpreted as a noticeable adaptation to cursoriality and running. Comparison with a biomechanical study of the foot of the ostrich (*Struthio camelus*) is particularly enlightening, because this extant bird is didactyl as result of adaptation towards fast and sustained bipedal locomotion^49^. Ostriches have lost digit II and display a raised metatarso-phalangeal pad. The very shallow digit II impression in *R*. *pozosaladensis* suggest a reduced role in body weight support, especially considering the moderate speed inferred for the producer. By comparison with didactyl ostriches, the functional advantage of this relative shortening probably lies in the lightening of the distal limb, with the consequent optimization of swing dynamics and potential higher stride frequencies^49^. A shallowing of digit II impression in running when compared to walking locomotion was also observed in the emu (*Dromaius novaehollandiae*)^50^, which is a large cursorial ratite species with tridactyl foot. The deep impressions of digit III in *R*. *pozosaladensis* indicate its function as main load-bearing digit, whereas the shallower and smaller lateral digit impression (IV) probably reflect its secondary role in body support and stabilizing the animal during rapid locomotion^49^. As in didactyl ostriches, large and deep claw imprint in digit III probably reflect an essential traction function during push-off, whereas reduced claw imprint in digit IV agrees with its function as outrigger^49^. The shallow impression of the metatarso-phalangeal pad in *R*. *pozosaladensis* is indicative of a secondary role as weight-bearing. Alternatively, this pad was potentially used during prey immobilization along with digit II claw as was observed in seriemas^14^ and has been also proposed for deinonychosaurs^51^. This inference is compatible with consumption of small prey or application of multi-targeted strikes on larger prey as suggested for the medium-sized Neogene phorusrhacid *Andalgalornis steulleti*^6^.

*Rionegrina pozosaladensis* reveals the presence of a phorusrhacid of moderate size, probably comparable to *M*. *milneedwardsi*, with noticeable adaptations for running and predation. The latter features include functionally didactyl posture, with stabilization during running facilitated by digit IV, and a combination of a large curved claw in digit II with an adjoining metatarso-phalangeal pad that assisted in the pinning and grasping of prey.

## Methods

### Geochoronology

U/Pb analyses were carried out at the R&D Productive Technology Center LA.TE.Andes (Salta, Argentina), with a combination of LA-ICP-MS instrumentation: a RESOlution 193 nm ablation laser fabricated by Australian Scientific Instruments and a triple quadrupole ICP-MS model 8900 fabricated by Agilent Technologies. Zircon grains for analysis were selected avoiding inclusions or fractures and measurements generally include one spot per grain analysed. The spot diameter used was 30 μm. The larger the spot diameter, the lower the fluence value selected^52^ and, in general, a lower frequency value, whose selection is based on the lowest relative standard dispersion (RSD) achieved during ICP-MS calibration in LA-ICP-MS mode. ICP-MS tuning is completed in two stages. In the first step, the sensitivity and stability in solution mode are optimized using an Agilent calibration solution containing 1 ppb of 7Li, 89Y and 205Tl. In the second stage, the calibration is done in LA-ICP-MS mode using a linear ablation (spot diameter and fluence according to those to be used in the sequence measurement, frequency between 5 and 10 Hz, scan speed of 1 μm/s) on NIST SRM 612 glass to reach a maximum intensity in cps (counts per second) at mass 238 so that 238U/232Th ≈ 1.05, 207Pb/206Pb ≈ 0.917, 208Pb/206Pb ≈ 2.17 and 248Th/232Th < 0.3% relationships are fulfilled, and with RSD ≤ 5%. A flow rate of 5 ml/min of N2 was added to favour the ionization of the elements in the plasma^53^. The He flow rate was 370 ml/min. Elemental concentrations are obtained considering NIST 610 glass as primary reference material (RM) and NIST 612 as secondary RM^54^. U/Pb ages are calculated from the isotopic ratios using 91500 zircon as reference material^55^ and repeated measurements on Plešovice zircon (reference TIMS age 337.13 ± 0.37 Ma)^56^ treated as control material/unknown sample. The laser ablation sequence is executed by measuring at the beginning and end of each sample three measurements of NIST SRM 612 and 610 glass, three of 91500 and two of Plešovice, with one analysis point of Plešovice and one of 91500 after every 20–30 analyses. The precision of the results is confirmed by obtaining the weighted average 238U/206Pb age of the Plešovice zircon. The analytical sequence included 25 s of background measurements (no ablation), followed by 25 s of ablation and 10 s of washout (laser off). LADR Software 1.1.0.7 was used for data reduction^57^. Subsequent processing was done with IsoplotR^58^.

### Photogrammetry

Fossil footprints were documented by photogrammetric techniques following the general procedure outlined in Falkingham^59^ and Mallison and Wings^60^. About 600 photos for the entire trackway were taken using a Nikon P510 camera and later processed using the software Agisoft PhotoScan Professional Edition (www.agisoft.com). Resultant models were scaled and oriented using the free software Meshlab (www.meshlab.net). Depth-colour images of the tridimensional models were obtained using the free software Paraview 5.0 (www.paraview.org).

### Ichnotaxonomy

Measurements on fossil footprints follow the general suggestions by Ref.^61^ and specific additional measurements and conventions are illustrated in Fig. 5. The morphological preservation scale and recommendations for proposal of new ichnotaxa follow Ref.^33^.

### Nomenclatural acts

This published work and the nomenclatural acts it contains have been registered in ZooBank, the proposed online registration system for the International Code of Zoological Nomenclature. The LSID for this publication is urn:lsid:zoobank.org:pub: F7D8E64D-B69C-42CE-8259-D2B2427BD9EB, and the LSIDs for the new erected taxa are: urn:lsid:zoobank.org:act:01DE373E-E2C8-4C67-B1CB-1946954112BB (*Rionegrina*) and urn:lsid:zoobank.org:act:629207A2-622E-4BA1-AF5A-E0D232E0B492 (*R*. *pozosaladensis*).

### Geometric morphometrics

Geometric morphometrics is an approach that allows the analysis of information on spatial covariation between anatomical landmarks^62^. The placement of landmarks assists in defining information on the shape of the track while excluding size. This makes an ideal means to assess tracks in an unbiased way, discounting size as a criterion and a means to understand shape and the sources of morphological variation. For this geometric morphometric analysis, 15 landmarks were digitally placed onto 39 tracks with good morphological preservation (i.e., distinguishable digits, clear track margins, etc.) from extant (*Rhea americana*, *Rhea pennata* and *Chunga burmeisteri*) and fossil birds (*Aramayoichnus rheae* and *Rionegrina pozosaladensis*). All photographs used were taken perpendicular to tracks, from the right side (either originally taken or mirrored). Landmarks were placed by a single operator to define the overall shape of the track. Landmark digitization utilized the freeware TPS Series (https://www.sbmorphometrics.org/), whereby TPS files were generated from images using tpsUtil and imported into tpsDig2 where all images were scaled and landmarks were digitized in the same order. Analysis of shape variation was then completed in the freeware program MorphoJ^63^ and landmarks were superimposed by Generalized Procrustes Analysis. Procrustes ANOVA, multivariate regression analysis, Principal Component Analysis (PCA), and Canonical Variate Analysis (CVA) of Procrustes shape coordinates were utilized to assess variation in shape.

### Supplementary Information

Supplementary Information.

## Data Availability

The datasets generated and/or analyzed during the current study are available in the Supplementary Material and Figshare.com repository. Orthomosaic (http://dx.doi.org/10.6084/m9.figshare.23749107), digital elevation model (http://dx.doi.org/10.6084/m9.figshare.23749260) and interpretative drawing (http://dx.doi.org/10.6084/m9.figshare.23751186) of complete described trackway including the holotype of *Rionegrina pozosaladensis* igen. et isp. nov. (footprints R1 to R6).
