# Supplementary material for: First terror bird footprints reveal functionally didactyl posture
Source: Sci Rep. 2023 Sep 30;13:16474. doi: 10.1038/s41598-023-43771-x (PMC10542783; doi:10.1038/s41598-023-43771-x)
Supplement: Supplementary file 1 — Supplementary Information. [file 41598_2023_43771_MOESM1_ESM.pdf]

Supplementary Material

First terror bird footprints reveal functionally didactyl posture

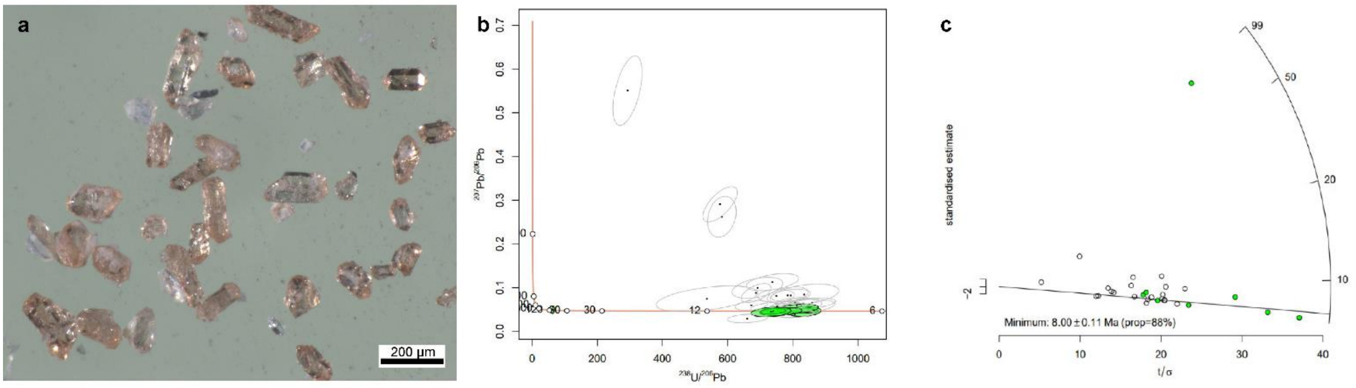

Fig. S1. U/Pb analyses on sample C893. a, Microscopic view of zircon picking**. b,** Tera-Wasserburg concordia diagram of zircon data (30 spots). Colourless ellipses represent discordant data and green ellipses represent concordant data. The concordance filter corresponds to values at 100 ± 10% with ^{206}Pb/^{238}U vs. ^{207}Pb/^{235}U ratio. **c**, Radial plot with minimum age model (tmin).

	Chemical content			Radiogenic ratios						Isotopic age (Ma)						Preferred age			Disc. %
Spot#	Th (ppm)	U (ppm)	Th/U (mass)	^{207}Pb/ ^{235}U	1s	^{206}Pb/ ^{238}U	1s	^{207}Pb/ ^{206}Pb	1s	^{206}Pb/ ^{238}U	1s	^{207}Pb/ ^{235}U	1s	^{207}Pb/ ^{206}Pb	1s	^{206}Pb/ ^{238}U	1s		(206/238)/ (207/235)
12	405.25	757.91	0.53	0.01059453	0.00123637	0.00116294	0.00006392	0.06491293	0.00797675	7.5	0.4	10.7	1.2	770.6	94.7	7.5	0.4	5%	70%
30	3345.88	2618.52	1.28	0.00783476	0.00065408	0.00118553	0.00003199	0.04676350	0.00359908	7.6	0.2	7.9	0.7	36.1	2.8	7.6	0.2	3%	96%*
05	144.80	316.15	0.46	0.01404437	0.00246090	0.00119645	0.00009993	0.08379147	0.01600426	7.7	0.6	14.2	2.5	1286.8	245.8	7.7	0.6	8%	54%
06	898.63	935.87	0.96	0.00757531	0.00122958	0.00119979	0.00005128	0.04571039	0.00747712	7.7	0.3	7.7	1.2	0.0	0.0	7.7	0.3	4%	101%*
18	660.71	972.34	0.68	0.01054545	0.00130929	0.00120152	0.00005469	0.06385782	0.00732565	7.7	0.4	10.7	1.3	736.0	84.4	7.7	0.4	5%	73%
16	7395.98	2248.63	3.29	0.00838480	0.00087255	0.00120603	0.00003637	0.04873122	0.00464638	7.8	0.2	8.5	0.9	133.9	12.8	7.8	0.2	3%	92%*
24	177.28	374.61	0.47	0.00910423	0.00159258	0.00120890	0.00009885	0.05723694	0.01061144	7.8	0.6	9.2	1.6	499.7	92.6	7.8	0.6	8%	85%
20	344.87	699.76	0.49	0.00871621	0.00114461	0.00122649	0.00006270	0.05171649	0.00708740	7.9	0.4	8.8	1.2	271.9	37.3	7.9	0.4	5%	90%*
23	377.14	716.33	0.53	0.00981905	0.00135750	0.00123154	0.00006710	0.05935341	0.01079807	7.9	0.4	9.9	1.4	579.1	105.4	7.9	0.4	5%	80%
27	348.55	671.92	0.52	0.01112948	0.00127776	0.00123943	0.00006076	0.06562737	0.00793142	8.0	0.4	11.2	1.3	793.6	95.9	8.0	0.4	5%	71%
19	540.66	758.62	0.71	0.00690803	0.00089396	0.00124082	0.00006078	0.03979429	0.00529944	8.0	0.4	7.0	0.9	0.0	0.0	8.0	0.4	5%	114%
17	326.25	647.20	0.50	0.00705550	0.00102591	0.00124382	0.00006095	0.04122998	0.00620584	8.0	0.4	7.1	1.0	0.0	0.0	8.0	0.4	5%	112%
03	300.57	646.07	0.47	0.01421014	0.00155341	0.00125949	0.00006264	0.08242323	0.00941422	8.1	0.4	14.3	1.6	1254.7	143.3	8.1	0.4	5%	57%
29	339.55	626.58	0.54	0.00918893	0.00117303	0.00126369	0.00007560	0.05291751	0.00705122	8.1	0.5	9.3	1.2	324.2	43.2	8.1	0.5	6%	88%
07	269.83	520.16	0.52	0.01411747	0.00198310	0.00127483	0.00006782	0.08260222	0.01259772	8.2	0.4	14.2	2.0	1259.0	192.0	8.2	0.4	5%	58%
02	292.76	551.59	0.53	0.00811268	0.00107844	0.00130899	0.00007362	0.04525619	0.00623449	8.4	0.5	8.2	1.1	0.0	0.0	8.4	0.5	6%	103%*
10	343.64	676.64	0.51	0.01022219	0.00135343	0.00133066	0.00009408	0.05604869	0.00777958	8.6	0.6	10.3	1.4	453.3	62.9	8.6	0.6	7%	83%
04	292.61	541.47	0.54	0.01536299	0.00187293	0.00133471	0.00006605	0.08143233	0.00961059	8.6	0.4	15.5	1.9	1231.0	145.3	8.6	0.4	5%	56%
14	902.73	928.63	0.97	0.00853645	0.00083095	0.00135121	0.00004638	0.04620153	0.00458553	8.7	0.3	8.6	0.8	7.1	0.7	8.7	0.3	3%	101%*
08	377.07	655.03	0.58	0.02134814	0.00222678	0.00135567	0.00009763	0.11263227	0.01288018	8.7	0.6	21.4	2.2	1841.5	210.6	8.7	0.6	7%	41%
28	370.94	704.88	0.53	0.00830456	0.00130819	0.00136317	0.00007506	0.04455572	0.00730852	8.8	0.5	8.4	1.3	0.0	0.0	8.8	0.5	6%	105%*
13	193.19	396.72	0.49	0.01937069	0.00279554	0.00144525	0.00010747	0.09991406	0.01587200	9.3	0.7	19.5	2.8	1621.7	257.6	9.3	0.7	7%	48%
25	568.11	801.44	0.71	0.01707393	0.00243746	0.00145404	0.00006341	0.08767800	0.01420042	9.4	0.4	17.2	2.5	1374.6	222.6	9.4	0.4	4%	54%
26	261.14	582.55	0.45	0.01208045	0.00147190	0.00148645	0.00007225	0.05949306	0.00759592	9.6	0.5	12.2	1.5	584.2	74.6	9.6	0.5	5%	79%
21	235.03	538.03	0.44	0.00615633	0.00131914	0.00151504	0.00009291	0.02939965	0.00648556	9.8	0.6	6.2	1.3	0.0	0.0	9.8	0.6	6%	157%
22	806.40	706.77	1.14	0.06215198	0.00724045	0.00171724	0.00008557	0.26241958	0.03082849	11.1	0.6	61.2	7.1	3260.6	383.0	11.1	0.6	5%	18%
15	500.56	879.43	0.57	0.06946469	0.00472452	0.00173371	0.00010485	0.29090176	0.02684098	11.2	0.7	68.2	4.6	3421.7	315.7	11.2	0.7	6%	16%
11	71.47	98.97	0.72	0.01624965	0.00390405	0.00186456	0.00035777	0.07464943	0.01941652	12.0	2.3	16.4	3.9	1058.2	275.2	12.0	2.3	19%	73%
01	390.50	299.36	1.30	0.26780920	0.02578520	0.00341201	0.00034392	0.55086127	0.05263143	22.0	2.2	240.9	23.2	4382.8	418.7	22.0	2.2	10%	9%
09	40.49	63.25	0.64	0.09834125	0.01222332	0.01554128	0.00065949	0.04913779	0.00604558	99.4	4.2	95.2	11.8	153.4	18.9	99.4	4.2	4%	104%*

Table S1. Results of U/Pb analyses of sample C893. * concordant data (100 ± 10) %.

Table S2. Measurements on *Rionegrina pozosaladensis* igen. et isp. nov.

Footprint #	MP	FL	FW	FL/FW	II	III	IV	w-II	w-III	w-IV	dIII	MTl	MTw	II-IV (°)	II-III (°)	III-IV (°)
R1	3	385	243	1.58	82	253	171	52	89	60	25	74	86	47.98	24.36	23.5
L1	3	385	254	1.52	90	276	171	48	90	65	35	nd	84	55.4	25.12	30.31
R2	3	372	242	1.54	100	255	165	50	87	72	32	77	78	53.18	26.34	26.45
L2	3	381	252	1.51	89	237	149	48	93	59	45	82	83	46.26	20.42	25.84
R3	3	397	244	1.63	88	270	176	49	89	64	40	71	84	49.29	23.68	25.47
L3	3	382	251	1.52	111	247	148	54	84	52	52	nd	78	49.24	23.03	26.77
R4	3	379	252	1.50	106	250	181	50	89	58	45	79	76	43.2	20.89	22.38
L4	3	322	252	1.28	67	228	160	39	89	59	40	65	63	57.87	28.34	29.29
R5	2	373	257	1.45	73	245	163	44	90	55	57	72	66	50.51	24.12	26.48
L5	2	336	284	1.18	67	244	148	26	87	59	40	66	56	53.4	26.32	26.68
R6	2	374	252	1.48	63	246	159	36	87	63	30	74	73	43.37	21.54	22.1
R7	2	323	261	nd	84	250	nd	46	90	58	35	nd	80	59.24	30.64	28.28
R8	2	nd	nd	nd	nd	206	170	nd	71	51	37	nd	nd	nd	nd	18.89
L8	2	nd	nd	nd	nd	235	129	nd	83	48	28	nd	nd	nd	nd	19.68
R9		nd	nd	nd	nd	nd	nd	49	89	nd	nd	nd	nd	nd	nd	nd
average		371.45	253.00	1.47	85.09	250.08	163.42	45.46	88.29	60.33	38	73.33	75.58	49.97	24.01	26.13
Std. dev		22.34	11.35	0.13	16.36	12.96	11.05	7.68	2.55	5.16	9	5.61	9.42	4.72	2.46	2.54
n		11	11	12	11	12	12	13	14	12	14	9	12	11	11	12
SE		6.74	3.42	0.04	4.93	3.74	3.19	2.13	0.68	1.49	2.40	1.87	2.72	1.42	0.74	0.73

Measurements in mm, except when stated. R1-R9, L1-L8: right and left consecutive footprints; FL: footprint length; FW: footprint width; II, III, IV: length of digits II to IV; w-II to w-IV: width of digits II to IV; dIII: maximum depth of digit III; MTl, MTw: length and width of metatarsal-phalangeal pad; II-IV: total divarication; II-III, III-IV: angle between digits II and III and between III and IV, respectively; nd: no data. See also Figure 5.

Table S2. (contd.) | Measurements on *Rionegrina pozosaladensis* igen. et isp. nov.

Footprint #	Cl-II	TCl-II	TCw-II	Cl-III	Cw-III	Cl-IV	Cw-IV	PA (°)	PL	PL/FL	SL	Fr (°)	Tbe	Bbt	Af (mm²)	W (g)
R1	77.3	13	10	70	29	23	38					-9.8			32757	54216
L1	72.7	15	15	77	35	30	22	162.28	892	2.32	1824	-6.7	452	nd	36715	62526
R2	66.7	13	9	63	30	35	38	174.25	935	2.51	1840	-10.5	365	-86	34836	58552
L2	72.5	13	12	69	38	38	31	174.65	930	2.44	1871	-5.6	371	-99	32740	54182
R3	79.1	12	10	nd	nd	37	25	163.46	953	2.40	1795	-7.7	448	-64	37116	63380
L3	69.4	9.6	15	78	38	29	21	165.78	865	2.26	1795	-5.6	414	-50	33490	55738
R4	66	10	13	81	40	22	25	167.44	940	2.48	1795	-7.1	409	-65	36051	61115
L4	74.7	17	9	64	29	30	22	167.15	901	2.80	1801	-6.6	418	-63	28707	45971
R5	nd	nd	nd	77	39	27	23	169.53	935	2.51	1876	-4.8	409	-80	32456	53594
L5	nd	nd	nd	77	28	35	29	171.94	912	2.71	1866	-6.2	385	-100	27293	43159
R6	nd	nd	nd	67	27	34	33	nd	944	2.52	1867	-5.5	nd	nd	31898	52445
R7	nd	nd	nd	nd	nd	nd	nd	nd	nd	nd	nd	-8	nd	nd	nd	nd
R8	nd	nd	nd	nd	nd	23	19	nd	nd	nd	nd	-4.7	335	nd	nd	nd
L8	nd	nd	nd	63	31	34	17	171.22	910	nd	1803	-6.7	337	nd	nd	nd
R9	nd	nd	nd	nd	nd	nd	nd	nd	901	nd	nd	nd	nd	nd	nd	nd
average	**72.30**	12.83	11.63	71.45	33.09	30.54	27.91	168.77	918.17	2.50	1830.27	-6.82	407.89	-75.88		54989
Std. dev	**4.73**	2.43	2.50	6.73	4.95	5.50	6.32	4.28	25.71	0.16	34.41	-1.72	30.46	-18.25		
n	**8**	8	8	11	11	13	11	10	12	10	11	14	9	8		
SE	**1.67**	0.86	0.89	2.03	1.49	1.53	1.90	1.35	7.42	0.05	10.38	-0.46	10.15	-6.45		

Cl-II: length of claw II; TCl-II, TCw-II: length and width of imprint of tip of claw II; Cl-III, Cw-III: length and width of claw of digit III; Cl-IV, Cw-IV: length and width of claw of digit IV; PA: pace angulation; PL: pace length; Fr: footprint rotation with respect to the midline; Tbe: external trackway breadth; Bbt: breadth between tracks (negative indicate footprint superposition); Af: area of footprint; W: body mass (W=10^(1.25 log Af – 0.91)[1]; nd: no data. See also Figure 5.

Species / ichnospecies	Locality/Source	File name
Rhea pennata	Cerro Chivo (43° 12' 48.56” S; 68° 50' 42.91” W), Chubut Province, Argentina.	RpCH1
Rhea pennata	Portezuelo Sumich (46° 48' 26.75” S; 70° 45' 38.74” W), Santa Cruz Province, Argentina.	RpPI1
Rhea pennata	La Amarga (39° 26' 59.76” S; 70° 10' 58.15” W), Neuquén Province, Argentina.	RpZA1
Rhea americana	Gran Salitral (37° 24’ 26.50” S, 67° 12’ 29.85” W), La Pampa Province, Argentina.	RaGS1
Rhea americana	Gran Salitral (37° 24’ 26.50” S, 67° 12’ 29.85” W), La Pampa Province, Argentina.	RaGS2
Rhea americana	Punta de la Barda (37° 09’ 43.31” S, 67° 12’ 40.11” W), La Pampa Province, Argentina.	RaBA1
Rhea americana	Punta de la Barda (37° 09’ 43.31” S, 67° 12’ 40.11” W), La Pampa Province, Argentina.	RaBA2
Rhea americana	Salitral de La Perra (37° 31’ 20.37” S, 67° 36’ 35.50” W), La Pampa Province, Argentina.	RaSP1
Rhea americana	Salitral de La Perra (37° 31’ 20.37” S, 67° 36’ 35.50” W), La Pampa Province, Argentina.	RaSP2
Rhea americana	Parque Nacional Talampaya (30° 07' 54.72" S; 67° 41' 40.19" W), La Rioja Province, Argentina.	RaTA1
Rhea americana	Laguna Salada (37° 06’ 04.35” S, 65° 27’ 23.43” W), La Pampa Province, Argentina.	RaVD1
Rhea americana	Laguna Salada (37° 06’ 04.35” S, 65° 27’ 23.43” W), La Pampa Province, Argentina.	RaVD2
Chunga burmeisteri	Parque Nacional Talampaya (29° 47' 13.70" S; 67° 49' 59.28" W), La Rioja Province, Argentina.	CbTA1
Chunga burmeisteri	Parque Nacional Talampaya (29° 47' 13.70" S; 67° 49' 59.28" W), La Rioja Province, Argentina.	CbTA2
Chunga burmeisteri	Parque Nacional Talampaya (29° 47' 13.70" S; 67° 49' 59.28" W), La Rioja Province, Argentina.	CbTA3
Chunga burmeisteri	Fig 6.32, Farlow et al.[2]	CbFA1
Aramayoichnus rheae	Fig. 14B, Aramayo et al.[3] (Late Pleistocene).	ArPC1
Aramayoichnus rheae	Fig. 14E, Aramayo et al.[3] (Late Pleistocene).	ArPC2
Aramayoichnus rheae	Fig. 4.87A, Feola[4] (Late Pleistocene).	ArPC3
Aramayoichnus rheae	Pehuen Co (39° 00' 06.07" S; 61° 31' 01.85" W), Buenos Aires Province, Argentina (Late Pleistocene).	ArPC4
Aramayoichnus rheae	Bajada El Faro (41°03' 55.81" S/ 62° 51' 16.98" W), Río Negro Province, Argentina (Late Miocene).	ArRN1
Aramayoichnus rheae	Fig. 2 (LFp1), Tineo et al.[5] (Late Miocene-Pliocene).	ArGF1
Aramayoichnus rheae	Fig. 2 (RFp2), Tineo et al.[5] (Late Miocene-Pliocene).	ArGF2
Aramayoichnus rheae	Fig. 2 (LFp3), Tineo et al.[5] (Late Miocene-Pliocene).	ArGF3
Aramayoichnus rheae	Quebrada de La Troya (28° 43' 42.80" S; 68° 15' 22.91" W), La Rioja Province, Argentina (Middle-Late Miocene).	ArFV1
Rionegrina pozosaladensis	R1, this study (Late Miocene).	RzPZ1
Rionegrina pozosaladensis	L1, this study (Late Miocene).	RzPZ2
Rionegrina pozosaladensis	R2, this study (Late Miocene).	RzPZ3
Rionegrina pozosaladensis	L2, this study (Late Miocene).	RzPZ4
Rionegrina pozosaladensis	R3, this study (Late Miocene).	RzPZ5
Rionegrina pozosaladensis	L3, this study (Late Miocene).	RzPZ6
Rionegrina pozosaladensis	R4, this study (Late Miocene).	RzPZ7
Rionegrina pozosaladensis	L4, this study (Late Miocene).	RzPZ8
Rionegrina pozosaladensis	Field photo, track of holotype, this study (Late Miocene).	RdPZ1
Rionegrina pozosaladensis	Field photo, track of holotype, this study (Late Miocene).	RdPZ2
Rionegrina pozosaladensis	Field photo, track of holotype, this study (Late Miocene).	RdPZ3
Rionegrina pozosaladensis	Field photo, track of holotype, this study (Late Miocene).	RdPZ4
Rionegrina pozosaladensis	Field photo, track of holotype, this study (Late Miocene).	RdPZ5
Rionegrina pozosaladensis	Field photo, track of holotype, this study (Late Miocene).	RdPZ6

Table S3. Extant and fossil footprints used in geometric morphometric analyses.

Table S4. Geometric morphometrics

New Procrustes fit:

15 landmarks in 2 dimensions.

The dataset contains 39 observations, of which 39 are included for analyses.

Average shape:

Lmk.	Axis 1 (x)	Axis 2 (y)
1	-0.24326348	0.12006309
2	-0.21111161	0.00639872
3	-0.12030606	-0.16674700
4	-0.02392634	0.45410604
5	-0.02877533	0.32467831
6	-0.00984962	-0.07238859
7	-0.09358358	0.12442327
8	0.05744092	0.12963357
9	0.00364010	-0.16536717
10	-0.06439652	-0.25400124
11	0.01005216	-0.33046426
12	0.08087036	-0.24485838
13	0.27218316	0.15109763
14	0.24640503	0.08190691
15	0.12462082	-0.15848089

Procrustes sums of squares: 0.6594354264268578

Tangent sums of squares: 0.6427229072820538

Principal Component Analysis: PCA: CovMatrix, newDataset, Procrustes coordinates

PC	Eigenvalues	% Variance	Cumulative %
1.	0.00469836	27.778	27.778
2.	0.00269849	15.954	43.733
3.	0.00233295	13.793	57.526
4.	0.00172744	10.213	67.739
5.	0.00121985	7.212	74.951
6.	0.00094944	5.613	80.565
7.	0.00070341	4.159	84.724
8.	0.00063273	3.741	88.464
9.	0.00048363	2.859	91.324
10.	0.00033712	1.993	93.317
11.	0.00026104	1.543	94.860
12.	0.00021742	1.285	96.146
13.	0.00015747	0.931	97.077
14.	0.00013445	0.795	97.872
15.	0.00010297	0.609	98.481
16.	0.00008583	0.507	98.988
17.	0.00006335	0.375	99.363
18.	0.00004206	0.249	99.611
19.	0.00002866	0.169	99.781
20.	0.00001683	0.099	99.880
21.	0.00000896	0.053	99.933
22.	0.00000449	0.027	99.960
23.	0.00000286	0.017	99.977
24.	0.00000212	0.013	99.989
25.	0.00000114	0.007	99.996
26.	0.00000069	0.004	100.000

Total variance: 0.01691376

Variance of the eigenvalues: 0.0000011767545

Eigenvalue variance scaled by total variance: 0.00411

Eigenvalue variance scaled by total variance and number of variables: 0.11123

Note: throughout all calculations of eigenvalue variances, the dimensionality used was 26.

Principal Component Coefficients

	PC1	PC2	PC3	PC4	PC5	PC6	PC7	PC8	PC9	PC10	PC11	PC12	PC13
x1	0.318059	0.345816	0.046066	-0.233402	0.134738	-0.074638	-0.205238	-0.107275	-0.137676	-0.277435	0.194405	-0.166540	0.049769
y1	0.348453	-0.115337	0.027779	-0.111968	0.439939	0.340457	0.283832	0.014384	-0.034349	0.075903	0.029228	0.161246	0.118709
x2	0.219573	0.247598	0.105514	-0.098472	0.165560	-0.112240	-0.035452	-0.034697	-0.089384	-0.082207	0.137890	0.106519	-0.248460
y2	0.139941	-0.044077	-0.502890	0.000568	-0.047879	0.324784	-0.080250	-0.116094	0.247658	-0.242990	-0.419486	-0.234601	-0.077207
x3	-0.085297	0.344743	-0.190648	-0.316985	-0.010801	-0.042928	0.448659	-0.201766	0.159848	0.286012	-0.098766	-0.232629	0.089196
y3	-0.026166	-0.237590	-0.304929	0.203365	0.210389	-0.061533	-0.021727	0.103507	-0.010946	-0.016231	0.470829	-0.103754	-0.198639
x4	0.059826	-0.036052	0.113542	0.113127	-0.120845	-0.135601	0.131128	0.068942	0.089288	-0.074952	-0.026006	0.290113	0.354120
y4	-0.107088	0.356149	0.385354	0.145426	0.128405	0.137946	0.007997	0.163086	0.216771	0.032080	0.090486	-0.380582	0.170387
x5	0.040717	-0.005608	0.108577	0.262776	-0.218512	0.025625	-0.013070	-0.027488	0.000524	0.142561	-0.018533	0.016496	0.027709
y5	-0.281888	0.303116	-0.119185	0.004190	-0.018474	-0.011015	-0.032336	-0.276421	-0.055931	-0.198901	0.159616	0.433214	-0.178114
x6	-0.094076	0.047211	-0.036401	0.145334	-0.099600	0.108919	-0.021543	-0.091107	-0.009748	0.147186	0.027150	-0.091189	-0.194324
y6	0.067302	-0.025957	-0.119932	-0.003412	0.078526	-0.285666	-0.076456	0.261631	-0.446647	0.333144	-0.275864	-0.155381	0.175827
x7	-0.148039	0.099490	-0.190731	0.244608	-0.391781	0.116747	-0.047397	0.043660	-0.102463	-0.000844	0.187068	-0.195847	0.157003
y7	-0.097945	0.134969	-0.114489	0.025456	-0.040784	-0.161758	-0.054684	0.005828	-0.261511	0.032463	-0.096094	0.097017	0.072626
x8	0.084756	-0.063235	0.225718	0.182367	0.019395	0.066410	-0.086496	-0.214127	-0.009555	0.308615	-0.278077	0.064677	-0.502411
y8	-0.132482	0.136816	-0.150022	-0.025075	0.047906	-0.120478	-0.048640	-0.028530	-0.300248	0.046010	-0.085130	0.078474	-0.043554
x9	-0.021500	-0.068292	-0.041673	0.093517	0.110415	0.107986	-0.073035	0.049367	0.019566	0.002065	-0.018421	0.133679	0.174454
y9	-0.166990	-0.144651	0.142668	0.080907	0.111047	-0.041709	-0.087196	-0.031402	0.114263	0.047918	0.272109	-0.261066	-0.025245
x10	-0.135904	-0.036453	-0.118117	-0.002070	-0.031668	0.393418	0.116063	0.247282	-0.135910	-0.128885	0.113953	0.167445	0.143677
y10	-0.168095	-0.086694	0.191747	-0.092480	0.033865	-0.146661	-0.299434	-0.220245	0.262841	-0.091305	-0.181238	0.039116	0.209053
x11	-0.058638	-0.071922	-0.119747	0.098409	0.219453	-0.051530	-0.259215	-0.149796	0.188542	-0.045238	-0.120033	0.164807	0.233777
y11	-0.229929	-0.049626	0.300337	-0.222731	-0.161664	0.357696	0.171083	0.139566	-0.153116	0.009113	-0.093422	0.156998	-0.117846
x12	0.061910	-0.140668	-0.006254	0.213699	0.274470	0.007428	0.000363	-0.050644	0.027487	0.246117	0.027894	0.046558	0.057199
y12	-0.216505	-0.099978	0.178263	-0.102427	0.026927	0.044279	-0.066489	-0.092608	0.021763	0.004198	-0.054448	-0.042541	-0.005474
x13	-0.195661	-0.354068	-0.092206	-0.398945	-0.030707	-0.171208	0.124975	-0.042272	0.072227	0.025284	0.165728	-0.140204	-0.186929
y13	0.369083	-0.299018	0.159061	0.031601	-0.364205	-0.063210	0.211848	-0.481851	-0.212385	-0.130316	0.128515	-0.093670	0.184764
x14	-0.039801	-0.206422	0.019906	-0.426338	-0.089522	-0.134843	-0.178277	0.254627	0.107023	0.018399	0.002901	0.067907	0.062887
y14	0.411630	0.098907	-0.021232	-0.146328	-0.361840	0.099248	-0.344137	0.310151	0.217660	0.187040	0.110276	0.116368	-0.150311
x15	-0.005924	-0.102136	0.176454	0.122376	0.069406	-0.103546	0.098537	0.255294	-0.179767	-0.566679	-0.297151	-0.231793	-0.217667
y15	0.090680	0.072971	-0.052531	0.212907	-0.082158	-0.412378	0.436591	0.249001	0.394178	-0.088126	-0.055377	0.189163	-0.134976

PC14	PC15	PC16	PC17	PC18	PC19	PC20	PC21	PC22	PC23	PC24	PC25	PC26
-0.126054	0.358326	-0.197175	-0.177974	-0.299176	0.141000	-0.076868	-0.041854	0.014462	-0.005597	-0.071574	-0.005528	0.061834
-0.310699	0.077026	0.237488	0.325934	0.002074	0.034687	0.011058	0.037680	0.045803	0.016068	0.052430	0.012795	-0.021920
0.318561	-0.654983	0.102887	0.221796	0.073147	-0.060008	0.058786	0.060429	-0.024921	-0.014723	0.033885	0.024831	-0.012048
0.107286	-0.237879	-0.124279	-0.148658	-0.116256	-0.036046	0.010791	-0.054060	-0.059435	-0.019942	-0.031005	0.028926	0.006000
-0.030133	0.121156	0.087863	-0.112276	0.350189	-0.186276	-0.045866	0.064960	-0.000417	0.002121	0.042722	0.006566	-0.059334
0.383679	0.292409	0.202079	-0.168777	0.201638	-0.121971	-0.038012	0.042679	0.043540	-0.014949	0.021222	-0.029161	-0.061698
0.082704	0.000808	-0.144764	-0.248720	0.226968	-0.059790	0.478546	-0.187848	0.015103	-0.021908	0.073351	0.001257	0.045577
0.322047	-0.015103	-0.000360	-0.029684	-0.082751	0.092267	0.037481	0.050318	0.007343	0.014144	-0.035091	-0.015721	0.021895
-0.016825	0.033247	-0.129391	0.123305	0.034130	0.006321	-0.495110	0.151322	-0.369204	-0.223719	0.104636	-0.038713	-0.414899
-0.163250	0.027370	0.011183	-0.056818	0.040966	-0.155368	0.018255	0.000189	-0.159226	0.071990	-0.399066	0.096983	-0.141225
-0.048509	0.044261	0.296541	0.104146	-0.098430	0.311949	0.1 125	-0.729626	-0.000713	-0.041145	0.052569	0.061368	-0.169614
0.039097	-0.040435	-0.003255	0.016549	0.000527	0.103833	0.117713	0.004020	-0.198329	0.133294	-0.439162	0.120811	-0.109682
-0.189631	-0.026165	0.231197	0.335952	-0.212239	-0.213829	0.167952	0.271173	0.160615	0.123449	-0.060071	-0.010024	0.247140
0.008717	-0.052416	-0.125219	0.033809	0.115761	0.068290	-0.330713	-0.089210	0.578392	-0.246604	0.251473	0.330026	0.144106
-0.017747	0.136058	0.055671	-0.242102	0.059200	0.172936	0.070735	0.303789	0.177357	0.139454	-0.080391	-0.034575	0.246288
-0.030606	0.019109	-0.019557	-0.025375	-0.106302	0.032246	0.134108	0.033654	-0.278989	0.047711	0.573982	-0.516496	0.127065
0.051936	-0.061773	-0.197782	-0.036130	-0.025052	-0.076173	-0.209959	-0.077157	0.314010	0.686377	0.042320	-0.190670	-0.313762
-0.527029	-0.333314	-0.168792	-0.229983	0.170876	0.063101	-0.004872	-0.016957	-0.172013	0.159511	0.155166	0.239344	0.096380
0.011659	-0.114191	-0.063275	-0.161537	0.199934	0.471850	-0.105943	0.124422	-0.043335	-0.226439	-0.208739	-0.193314	0.192555
0.061988	0.082197	0.178181	0.283498	0.240572	0.032313	-0.227047	-0.146835	-0.025813	0.026062	-0.190343	-0.298293	0.293169
0.125827	0.171264	0.063964	0.137972	-0.063881	0.147440	0.187400	0.265365	-0.191865	-0.038601	0.236470	0.469448	-0.053655
0.242974	0.147503	-0.090238	-0.008571	-0.211127	-0.226617	-0.045598	-0.088844	-0.163675	0.135758	0.108864	0.293904	0.130000
-0.034625	-0.056744	-0.245008	-0.055557	-0.246320	-0.493286	-0.070948	-0.222898	-0.029390	-0.338748	-0.163133	-0.116848	0.264337
-0.096528	-0.042735	-0.029736	-0.039007	-0.167874	-0.018864	0.311687	0.196673	0.351842	-0.364590	-0.051866	-0.219686	-0.511012
0.105674	0.038581	-0.417011	0.363698	-0.058040	0.175656	0.089496	0.014632	0.003064	0.035815	-0.064436	-0.007745	0.052427
0.138602	-0.064328	0.089396	-0.110891	-0.021327	0.043213	-0.028925	0.014976	-0.014909	0.040522	-0.015715	0.011260	0.003089
-0.067024	-0.133808	0.492563	-0.355497	-0.222356	-0.109287	-0.196185	0.035254	-0.009107	-0.037542	0.038685	0.020306	-0.021489
-0.094828	0.129156	-0.163199	0.132586	0.269947	-0.109914	0.131022	-0.033540	0.014065	-0.042915	0.040408	-0.024470	-0.036945
-0.165814	0.143962	0.063720	0.102922	0.281926	-0.228503	0.021839	-0.031965	-0.015659	-0.038794	0.023707	0.013641	-0.065356
-0.081450	0.011440	0.006307	0.025388	-0.336725	0.198830	-0.096948	0.049256	0.031404	0.043939	-0.041298	-0.030222	0.060778

Procrustes ANOVA

Classifiers used for the Procrustes ANOVA:

Individuals: species

Centroid size:

**Effect**	**SS**	**MS**	**df**	**F**	**P (param.)**
Individual	7254.516991	1813.629248	4	54.03	<.0001
Residual	1141.328558	33.568487	34		

Shape. Procrustes ANOVA:

Effect	SS	MS	df	F	P (param.)	Pillai tr.	P (param.)
Individual	0.20028257	0.0019257939	104	3.85	<.0001	3.53	<.0001
Residual	0.44244034	0.0005004981	884				

Regression Analysis

Log Centroid Size

Total sample size: 39

Regression Coefficients

	CentSize2
x1	0.02083332
y1	0.03027312
x2	0.01756994
y2	0.00611040
x3	-0.01856176
y3	-0.00251948
x4	-0.00012735
y4	-0.00158020
x5	0.00234437
y5	-0.02803160
x6	-0.00597767
y6	0.00861566
x7	-0.01904562
y7	-0.00984170
x8	0.01450584
y8	-0.01189082
x9	0.00067535
y9	-0.00822702
x10	-0.01123933
y10	-0.00526647
x11	-0.00064635
y11	-0.01443501
x12	0.00811242
y12	-0.01246750
x13	-0.01930856
y13	0.01439611
x14	0.00541604
y14	0.03221311
x15	0.00544937
y15	0.00265142

Sums of squares

Total SS: 0.64272291

Predicted SS: 0.06682090

Residual SS: 0.57590201

% predicted: 10.3965%

Permutation test against the null hypothesis of independence

Number of randomization rounds: 10000

P-value: 0.0002

Canonical Variate Analysis: CVA

Classification criterion: species

Ar: *Aramayoichnus rheae*, **Cb**: *Chunga burmeisteri*, **Ra**: *Rhea americana*, **Rp**: *Rhea pennata*, **Rz**: *Rionegrina pozosaladensis*

Groups	Observations
Ar	9
Cb	4
Ra	9
Rp	3
Rz	14

Variation among groups, scaled by the inverse of the within-group variation

	Eigenvalues	% Variance	Cumulative %
1.	63.00592213	56.567	56.567
2.	39.86309191	35.789	92.356
3.	6.06101313	5.442	97.797
4.	2.45348021	2.203	100.000

Mahalanobis distances among groups:

	Ar	Cb	Ra	Rp
Cb	18.7108			
Ra	11.4985	27.7126		
Rp	11.5464	21.5787	14.2005	
Rz	14.1293	22.1610	14.4853	8.4631

P-values from permutation tests (10000 permutation rounds) for Mahalanobis distances among groups:

	Ar	Cb	Ra	Rp
Cb	0.0005			
Ra	<.0001	0.0004		
Rp	0.0010	0.0092	0.0002	
Rz	<.0001	0.0001	<.0001	0.0007

Procrustes distances among groups:

	Ar	Cb	Ra	Rp
Cb	0.1120			
Ra	0.0796	0.1229		
Rp	0.1090	0.1450	0.0938	
Rz	0.1378	0.1411	0.1059	0.1167

P-values from permutation tests (10000 permutation rounds) for Procrustes distances among groups:

	Ar	Cb	Ra	Rp
Cb	0.0454			
Ra	0.0997	0.0002		
Rp	0.1668	0.0298	0.1922	
Rz	<.0001	0.0001	<.0001	0.0027

Canonical coefficients:

	CV1	CV2	CV3	CV4
x1	58.7995	59.8549	-23.0405	20.0161
y1	-36.5953	19.4189	-11.8044	-34.5755
x2	4.0034	30.4190	4.8751	6.8477
y2	123.1039	-5.8884	3.1594	26.0202
x3	-173.7507	-66.4136	-7.7500	9.1846
y3	-73.5385	-63.6802	50.2128	-25.1027
x4	-106.8261	-43.5839	-120.0964	39.2516
y4	17.0166	26.8758	-0.5114	26.7888
x5	386.4503	-270.6489	297.7573	26.9183

y5	268.5671	7.4822	-95.1551	152.0716
x6	275.1664	-51.7376	-16.3903	-87.6035
y6	375.4056	105.5124	-130.8917	171.8225
x7	-209.5027	80.1494	-110.2142	12.3372
y7	-37.0751	113.4116	-243.4509	-110.7808
x8	-183.3229	188.8019	-18.8340	3.9440
y8	-542.4209	-246.7846	439.7802	-214.0746
x9	-775.0424	-107.3115	217.9782	-210.8287
y9	191.6065	-5.9289	-180.0651	6.6887
x10	251.7591	58.6222	-27.2757	43.8132
y10	-181.6415	170.8915	21.4459	-109.8637
x11	385.2755	-118.4988	-168.4104	89.8649
y11	57.0407	35.2433	-149.3039	-19.0428
x12	39.4573	230.1645	-36.2617	57.4826
y12	-65.5164	-182.9427	275.0078	147.6124
x13	-104.7845	32.0354	21.1624	41.4001
y13	-18.9419	-1.0619	-26.8214	10.0811
x14	40.8396	28.3876	-4.5114	-63.1756
y14	-39.4568	-44.2668	-2.3549	-1.6668
x15	111.4785	-50.2407	-8.9883	10.5475
y15	-37.5540	71.7178	50.7525	-25.9785

Taxa	Provenance	Body mass	Age
Hinasuri nehuensis	Monte Hermoso Fm, Buenos Aires Province	Larger than *Rhea americana,* 38.25 kg - 35.77 kg[6]	Early Pliocene
Heterorhea dabbenei	Monte Hermoso Fm, Buenos Aires Province	Larger than *Rhea americana*[7]	Early Pliocene
Rhea mesopotamica	Ituzaingó Fm, Entre Ríos Province	Comparable to extant *R. pennata*[8]	Late Miocene
Pterocnemia?	Cerro Azul Fm, La Pampa Province	Unknown[9]	Late Miocene
Ophistodactylus kirchneri	Andalhualá Fm, Catamarca Province	10 % larger than *R. americana*[10]	Late Miocene-Early Pliocene
Rhea cf. *mesopotamica*	Aisol Fm, Mendoza Province	Gracile[8]	Early-Middle Miocene
Opistodactylus horacioperezzi	Chichinales Fm, Río Negro Province	Much smaller than *Heterorhea*[11]	Early Miocene
Opisthodactylus patagonicus	Santa Cruz Fm, Santa Cruz Province	Similar to *R. americana*[12]	Early Miocene
Diogenornis fragilis	Itaboraí Fm, Brazil	Gracile, much smaller than extant rheas[13]	Early Eocene

Table S5. Estimated body mass from Neogene records of Rheidae from South America (mostly from Argentina except when indicated).

Taxa	Provenance	Body mass	Age
Mesembriornis milneedwardsi	Monte Hermoso Fm, Buenos Aires Province	53.84 kg[14], 66 kg[15]	Early Pliocene
Devincenzia pozzi	Ituzaingó Fm, Entre Ríos Province	161.95 kg[15]	Late Miocene - Pliocene
Procariama simplex	Andalhualá Fm, Catamarca Province and Cerro Azul Fm, La Pampa Province	9.7-12.66 kg[16], 9.48 kg[14]	Late Miocene
Psilopterus colzecus	Cerro Azul Fm, La Pampa Province	7.8-10.24 kg[16]	Late Miocene
Mesembriornis cf. *milneedwardsi*	Andalhualá Fm, Catamarca Province	Médium-sized[17]	Late Miocene
Andalgalornis steulleti	Andalhuala Fm, Catamarca Province	30.8 kg[15]	Late Miocene
Mesembriornis incertus	Andalhuala Fm, Catamarca Province	30.8 kg[15]	Late Miocene
Psilopterus bachmanni	Santa Cruz Fm, Santa Cruz Province	9 kg[16], 4.5 kg[14]	Early-Middle Miocene (19-15 Ma)
Psilopterus lemoinei	Santa Cruz Fm, Santa Cruz Province	7 kg[18], 8.45 kg[16], 8.06 kg[14]	Early-Middle Miocene (19-15 Ma)
Patagornis marshi	Santa Cruz Fm, Santa Cruz Province	26.15 kg[14], 31.6 kg[15]	Early-Middle Miocene (19-15 Ma)
Phorusrhacos longissimus	Santa Cruz Fm, Santa Cruz Province	140 kg[14], 117 kg[15]	Early-Middle Miocene (19-15 Ma)
Psilopterus affinis	“Deseado Fm”, Chubut Province	9.92 kg[16]	Late Oligocene

Table S6. Stratigraphic distribution and estimated body mass of Oligocene-Pliocene Phorusrhacidae from Argentina.

References

1 Tanaka, I. Estimating body weight and habitat type from extinct avian and avian-like theropod footprints. *Lethaia* **48**, 188-195, doi:https://doi.org/10.1111/let.12098 (2014).

2 Farlow, J. O., Coroian, D. A. N. & Currie, P. J. *Noah's Ravens: Interpreting the Makers of Tridactyl Dinosaur Footprints*. (Indiana University Press, 2018).

3 Aramayo, S. A., Manera de Bianco, T., Bastianelli, N. V. & Melchor, R. N. Pehuen Co: Updated taxonomic review of a late Pleistocene ichnological site in Argentina. *Palaeogeogr Palaeocl* **439**, 144-165, doi:https://doi.org/10.1016/j.palaeo.2015.07.006 (2015).

4 Feola, S. F. *Iconología y Sedimentología del Yacimiento Paleoicnológico de Pehuen Co (Pleistoceno Tardío)* PhD thesis, Universidad Nacional del Sur, (2022).

5 Tineo, D. E. *et al.* The oldest record of *Aramayoichnus rheae* from the Neogene of northwestern Argentina. *Ameghiniana* **55**, 109-116, doi:10.5710/AMGH.01.08.2017.2989 (2018).
6 Picasso, M. B. J. & Mosto, M. C. New insights about *Hinasuri nehuensis* (Aves, Rheidae, Palaeognathae) from the early Pliocene of Argentina. *Alcheringa* **40**, 244-250, doi:10.1080/03115518.2016.1122961 (2016).
7 Rovereto, C. Los estratos araucanos y sus fósiles. *Anales del Museo de Historia Natural* **25**, 1–247. (1914).
8 Agnolin, F. & Noriega, J. I. Una nueva especie de ñandú (Aves: Rheidae) del Mioceno Tardío de la Mesopotamia Argentina. *Ameghiniana* **49**, 236-246, doi:10.5710/AMGH.v49i2(492) (2012).
9 Cenizo, M. M., Tambussi, C. P. & Montalvo, C. I. Late Miocene continental birds from the Cerro Azul Formation in the Pampean region (central-southern Argentina). *Alcheringa* **36**, 47-68, doi:10.1080/03115518.2011.582806 (2012).
10 Noriega, J. I., Jordan, E. A., Vezzosi, R. I. & Areta, J. I. A new species of *Opisthodactylus* Ameghino, 1891 (Aves, Rheidae), from the late Miocene of northwestern Argentina, with implications for the paleobiogeography and phylogeny of rheas. *J Vertebr Paleontol* **37**, e1278005, doi:10.1080/02724634.2017.1278005 (2017).
11 Agnolin, F. L. & Chafrat, P. New fossil bird remains from the Chichinales Formation (Early Miocene) of northern Patagonia, Argentina. *Annales de Paléontologie* **101**, 87-94, doi:https://doi.org/10.1016/j.annpal.2015.02.001 (2015).
12 Degrange, F. J., Noriega, J. I. & Areta, J. I. in *Early Miocene Paleobiology in Patagonia: High-Latitude Paleocommunities of the Santa Cruz Formation* (eds M. Susana Bargo, Richard F. Kay, & Sergio F. Vizcaíno) 138-155 (Cambridge University Press, 2012).
13 Alvarenga, H. Uma gigantesca ave fóssil do Cenozóico brasileiro: *Physornis brasiliensis* sp. n. *Anais da Academia Brasileira de Ciências* **54**, 697-712 (1982).
14 Degrange, F. J. & Tambussi, C. P. Re-examination of *Psilopterus lemoinei* (Aves, Phorusrhacidae), a late early Miocene little terror bird from Patagonia (Argentina). *J Vertebr Paleontol* **31**, 1082-1092, doi:10.1080/02724634.2011.595466 (2011).
15 Degrange, F. J. *Morfología del cráneo y complejo apendicular en aves fororracoideas: implicancias en la dieta y modo de vida* PhD thesis, Universidad Nacional de La Plata, (2012).
16 Vezzosi, R. I. First record of *Procariama simplex* Rovereto, 1914 (Phorusrhacidae, Psilopterinae) in the Cerro Azul Formation (Upper Miocene) of La Pampa Province; remarks on its anatomy, palaeogeography and chronological range. *Alcheringa: An Australasian Journal of Palaeontology* **36**, 157-169, doi:10.1080/03115518.2011.597657 (2012).
17 Vezzosi, R. I. & Noriega, J. About the systematic status of an old and forgotten specimen of terror bird (Phorusrhacidae: Mesembriornithinae) from the Miocene of Northwestern Argentina. *Contribuciones del Museo Argentino de Ciencias Naturales Bernardino Rivadavia* **7**, 69-77 (2018).
18 Alvarenga, H. M. F. & Höfling, E. Systematic revision of the phorusrhacidae (Aves: Ralliformes). *Papeis Avulsos de Zoologia* **43**, 55-91, doi:10.1590/S0031-10492003000400001 (2003).
